# Distinct BOLD variability changes in the default mode and salience networks in Alzheimer’s disease spectrum and associations with cognitive decline

**DOI:** 10.1038/s41598-020-63540-4

**Published:** 2020-04-15

**Authors:** Liwen Zhang, Xi-Nian Zuo, Kwun Kei Ng, Joanna Su Xian Chong, Hee Youn Shim, Marcus Qin Wen Ong, Yng Miin Loke, Boon Linn Choo, Eddie Jun Yi Chong, Zi Xuen Wong, Saima Hilal, Narayanaswamy Venketasubramanian, Boon Yeow Tan, Christopher Li-Hsian Chen, Juan Helen Zhou

**Affiliations:** 1 Department of Pharmacology, National University of Singapore, Singapore, Singapore; 2 Centre for Sleep and Cognition, Department of Medicine, Yong Loo Lin School of Medicine, National University of Singapore, Singapore, Singapore; 3 Memory Ageing and Cognition Centre, National University Health System, Singapore, Singapore; 4 Centre for Cognitive Neuroscience, Neuroscience and Behavioural Disorders Program, Duke-National University of Singapore Medical School, Singapore, Singapore; 5 Research Centre for Lifespan Development of Mind and Brain (CLIMB), Institute of Psychology, Chinese Academy of Sciences, Beijing, China; 6 Raffles Neuroscience Centre, Raffles Hospital, Singapore, Singapore; 7 St. Luke’s Hospital, Singapore, Singapore; 8 Clinical Imaging Research Centre, Yong Loo Lin School of Medicine, National University Health System, Singapore, Singapore

**Keywords:** Alzheimer's disease, Translational research, Alzheimer's disease

## Abstract

Optimal levels of intrinsic Blood-Oxygenation-Level-Dependent (BOLD) signal variability (variability hereafter) are important for normative brain functioning. However, it remains largely unknown how network-specific and frequency-specific variability changes along the Alzheimer’s disease (AD) spectrum and relates to cognitive decline. We hypothesized that cognitive impairment was related to distinct BOLD variability alterations in two brain networks with reciprocal relationship, i.e., the AD-specific default mode network (DMN) and the salience network (SN). We examined variability of resting-state fMRI data at two characteristic slow frequency-bands of slow4 (0.027–0.073 Hz) and slow5 (0.01–0.027 Hz) in 96 AD, 98 amnestic mild cognitive impairment (aMCI), and 48 age-matched healthy controls (HC) using two commonly used pre-processing pipelines. Cognition was measured with a neuropsychological assessment battery. Using both global signal regression (GSR) and independent component analysis (ICA), results generally showed a reciprocal DMN-SN variability balance in aMCI (vs. AD and/or HC), although there were distinct frequency-specific variability patterns in association with different pre-processing approaches. Importantly, lower slow4 posterior-DMN variability correlated with poorer baseline cognition/smaller hippocampus and predicted faster cognitive decline in all patients using both GSR and ICA. Altogether, our findings suggest that reciprocal DMN-SN variability balance in aMCI might represent an early signature in neurodegeneration and cognitive decline along the AD spectrum.

## Introduction

Alzheimer’s disease (AD) is the major cause of dementia, and increasing attention has been focused on early disease detection/prevention. Therefore, studying brain changes along the AD disease continuum is important, i.e., from normal aging to the prodromal stage (amnestic mild cognitive impairment, aMCI) and finally to dementia stage. Using resting-state functional connectivity methods^1–5^ that quantifies the temporal synchrony between brain regions, both AD and aMCI have been found to target large-scale networks, including reduced default mode network (DMN) connectivity and increased salience network (SN)^6–8^ connectivity, as well as aberrant connectivity between networks^9^ in AD, and disturbed connectivity in aMCI, especially in relation to the DMN^10–12^. However, resting-state functional connectivity cannot provide information about the temporal variability of blood oxygen-level-dependent (BOLD) signal amplitude. Notably, the human brain features inherently moment-to-moment signal variation, which is not just neural noise but functional and adaptive^2,13^. Nonetheless, the resting-state BOLD signal variability (variability hereafter) pattern along the AD disease continuum remains largely unclear.

Although still under discussion, variability has been suggested to reflect the complexity and information capacity of the neural systems^14^ and possibly correlates with balance between dynamical integration and segregation in brain areas/networks (i.e. metastability)^15^, which contributes to optimal brain functioning. Indeed, in healthy young individuals (whose brains are assumed to be optimal), variability has been associated with response speed and transition from fixation to cognitive-demanding tasks^16–18^. In a most recent study, increased variability in the SN including the insula and decreased variability in most of the other brain regions have been found across life span^19^. Moreover, generally reduced variability has been found in healthy elderly compared with healthy young individuals^20^, and also in neuropsychiatric disorders (e.g., traumatic brain injury, psychosis and bipolar disorder)^21–23^. These results suggest that variability could be a promising and effective measure to reflect disturbed brain functioning.

Despite its potential in revealing network complexity/metastability, variability has been understudied in AD and aMCI, and the results were inconsistent. Compared with healthy individuals, AD has shown reduced variability especially in the posterior DMN^24–26^, increased variability in different areas across studies such as the parahippocampal gyrus/hippocampus, superior frontal gyrus, temporal gyrus, supplementary motor area and postcentral gyrus^24,26^, or no altered variability^27^. In aMCI, a recent meta-analysis of 12 resting-state fMRI studies reported altered variability in widespread areas compared with healthy individuals, such as decreased variability in areas that belong to the DMN and the SN, as well as increased variability in the visual network and the hippocampus^28^.

Moreover, few studies have investigated the frequency-dependent variability pattern in AD or aMCI. Within the commonly investigated low-frequency band (0.009–0.08 Hz) especially in resting-state studies^2^, slow4 (0.027–0.073 Hz) and slow5 (0.01–0.027 Hz) explain primary slow oscillations in grey matter hemodynamic signals, and have strongest oscillations in the basal ganglia and anterior DMN respectively in healthy young individuals^29^, which possibly contributes to different neural processing^30^. Previous work mostly measured frequency-specific variability using amplitude of low-frequency fluctuations (ALFF) and/or fractional ALFF (fALFF) indices; the findings in AD were limited and inconsistent. Briefly, Veldsman and colleagues found that AD had increased slow4 variability in the DMN/visual network and slow 5 variability in the precentral/postcentral gyrus, while decreased slow4 variability in the temporal pole^25^. Nevertheless, another study reported increased slow4/5 variability in the temporal regions as well as increased slow5 and reduced slow4 variability in the basal ganglia in AD compared with controls^24^. Additionally, there were lower level of variability in the posterior DMN at both slow4 and slow5 in aMCI than that in healthy elderly^31^. These inconsistent results might imply that a new index for variability is needed.

While previous studies have provided important preliminary results of variability alterations in patients with cognitive impairment, their limitations in frequency-dependent investigation, different pre-processing pipelines, variability calculation methods, a lack of direct comparison between AD and aMCI, and relatively small sample size preclude them from reaching a convincing conclusion. Moreover, to our best knowledge, there has been no study on how frequency-specific variability relates to cognitive decline over time in AD spectrum.

In view of these gaps, we aimed to investigate frequency-dependent BOLD variability during resting-state in a large sample of AD, aMCI, and age-matched healthy controls (HC) and evaluated their relationships with cognitive impairment and decline. Given previous inconsistent results using ALFF/fALFF^24,25,31^, we employed a new variability index defined as standard deviation (SD) of the BOLD signal^16,17,20,21^. This SD-based variability index is a direct measure of BOLD signal fluctuation and has not been examined in AD/MCI. Importantly, we aimed to reveal consistent BOLD variability patterns using two commonly used fMRI pre-processing approaches, including global signal regression (GSR) and independent component analysis (ICA). Based on previous evidence of divergent DMN-SN network disruptions in AD^7,8^ and aMCI^32^, we hypothesized that compared with age-matched controls, AD group would show lower DMN variability and higher SN variability while aMCI group would show similar trend with lesser extent. We also sought to test if such variability changes would be related to neurodegeneration and cognitive performance at baseline and cognitive decline over time.

## Results

### Comparisons of variability patterns between AD, aMCI, and HC at slow5

With GSR, there was a main effect of group mainly in the SN/subcortical areas (i.e., insula, rolandic operculum, amygdala, putamen), medial temporal lobe (hippocampus and parahippocampal gyrus), visual network (VN) (i.e. lingual gyrus/fusiform gyrus), dorsal attention network (i.e. postcentral gyrus), as well as the DMN (angular gyrus) (Supplementary Table 1).

**Figure 1:**
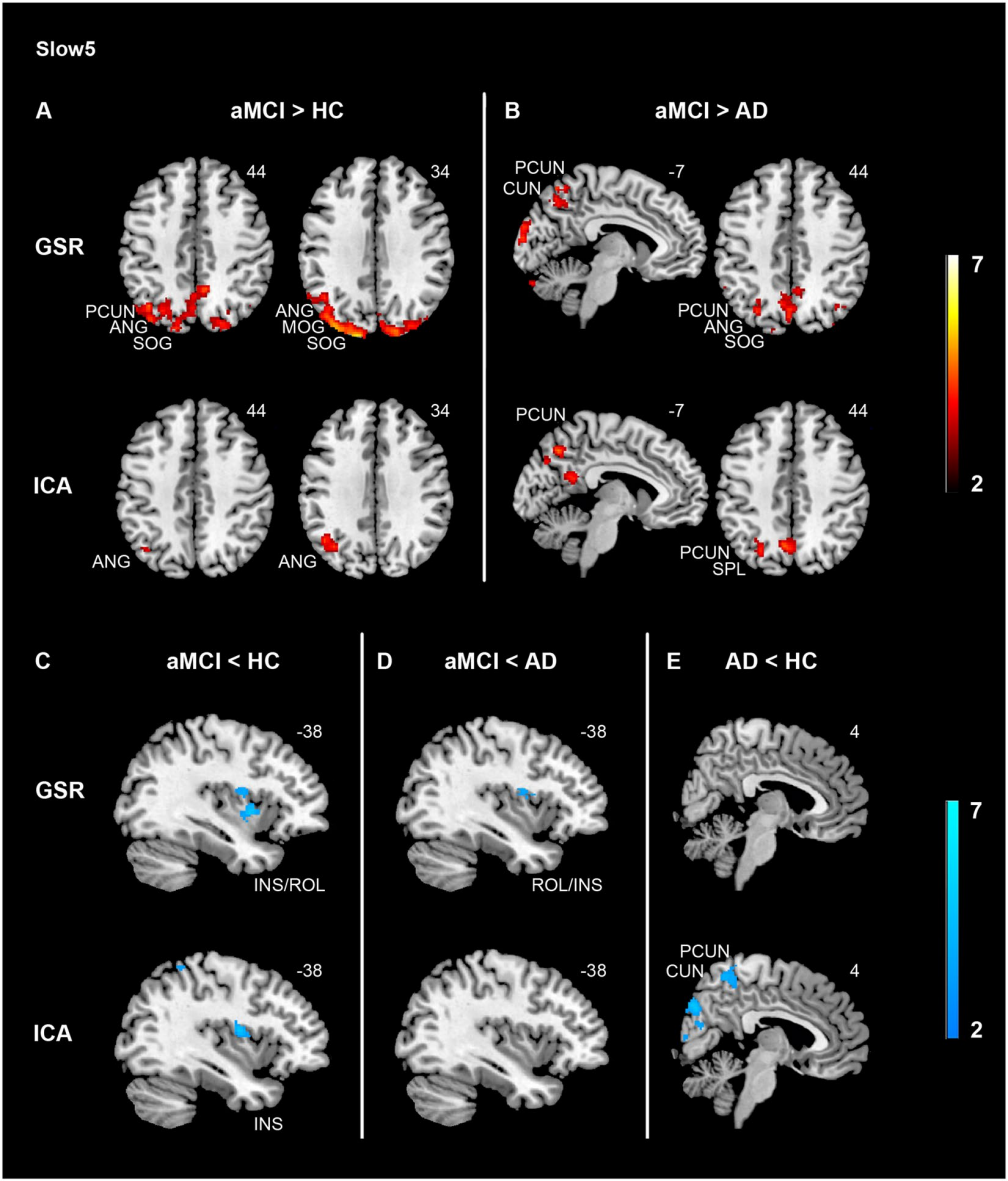
Divergent slow5 hemodynamic variability changes in the default mode and the salience networks in aMCI and AD. Using both GSR and ICA-based denoising methods, aMCI showed higher variability in the default mode network compared with HC (A, top panel) and AD (B, top panel), and lower variability in the salience network (INS) compared with HC (C, top panel). Specific to data denoising approaches, ICA-based denoising revealed lower variability in the default mode network (PCUN) in AD compared with HC (E, bottom panel), while GSR method showed lower variability in the salience network (INS) in aMCI compared with AD (D, top panel). Results were obtained at p < 0.05 family wise error (FWE) correction on the cluster level, with a previous height threshold of p < 0.001, superimposing on the MNI brain template. We also reported results with a less stringent cluster-level threshold of p < 0.05 (uncorrected, k > 40), with a previous height threshold of p < 0.001 (for the insula at aMCI < HC [C, bottom panel] with ICA-based denoising). Same slices were displayed to ease comparison between GSR approach and ICA-based denoising. Colour bar represents T value. Abbreviations: AD = Alzheimer’s disease; aMCI = Amnestic mild cognitive impairment; ANG = Angular gyrus; CUN = Cuneus; GSR = Global signal regression; HC = Healthy controls; ICA = Independent component analysis; INS = Insula; PCUN = Precuneus; ROL = Rolandic operculum; SOG = Superior occipital gyrus.

Pair-wise comparisons showed that there was increased variability in the posterior DMN/VN (precuneus, angular gyrus, cuneus, middle and superior occipital gyrus) in aMCI compared with HC (Fig. 1A, top panel). Similarly, aMCI mainly had higher variability in the posterior DMN/VN compared with AD (Fig. 1B, top panel), including the lingual gyrus, fusiform gyrus, angular gyrus, precuneus, cuneus, superior, middle and inferior occipital gyrus. In contrast, decreased variability was found in the SN in aMCI compared with both HC (Fig. 1C, top panel) and AD (Fig. 1D, top panel). Additionally, aMCI showed lower variability in the amygdala/hippocampus as well as the putamen compared with AD, and reduced variability in the parahippocampal gyrus and putamen compared with HC (Supplementary Table 1).

After ICA-based denoising, there was a main effect of group mainly in the posterior DMN including the precuneus/posterior cingulate cortex and VN (cuneus), and the SN (insula) (Supplementary Table 1). Group comparisons replicated the GSR findings that aMCI had higher variability in the posterior DMN compared with AD (precuneus; Fig. 1B, bottom panel) and HC (angular gyrus; Fig. 1A, bottom panel), as well as lower SN variability compared with HC (insula; Fig. 1C, bottom panel), although the latter did not survive the cluster-level FWE correction. Moreover, AD showed lower variability in the posterior DMN (i.e., precuneus/cuneus) than HC, which was absent using GSR approach (Fig. 1E).

### Comparisons of variability patterns between AD, aMCI, and HC at slow4

**Figure 2:**
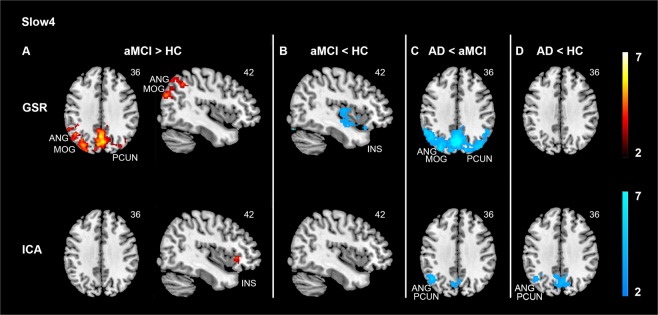
Divergent slow4 hemodynamic variability changes in the default mode and the salience networks in aMCI and AD. Compared with HC, GSR approach revealed higher variability in the default mode network (A, top panel) while lower variability in the salience network (B, top panel) in aMCI. However, after ICA-based denoising, there was higher variability in the salience network in aMCI than in HC (A, bottom panel). Moreover, AD showed lower variability in the default mode network (PCUN, ANG) compared with HC after ICA-based denoising (D, bottom panel), which was absent for the GSR approach (D, top panel). Across both data denoising methods, AD showed lower variability in the default mode network compared with aMCI (C). Results were obtained at p < 0.05 family wise error (FWE) correction on the cluster level, with a previous height threshold of *p* < 0.001, superimposing on the MNI brain template. Same slices were displayed to ease comparison between GSR approach and ICA-based denoising. Colour bar represents T value. Abbreviations: AD = Alzheimer’s disease; aMCI = Amnestic mild cognitive impairment; ANG = Angular gyrus; GSR = Global signal regression; HC = Healthy controls; ICA = Independent component analysis; INS = Insula; MOG = Middle occipital gyrus; PCUN = Precuneus.

With GSR, there was a main effect of group in the DMN including the precuneus and the angular gyrus, the SN including the insula, as well as the VN including the middle occipital gyrus and cuneus (Supplementary Table 2). Group comparisons revealed that aMCI had higher variability in the posterior DMN extending into the VN compared with both HC (Fig. 2A, top panel) and AD (Fig. 2C, top panel), whereas aMCI showed lower variability in the SN including the right insula (Fig. 2B, top panel). In parallel, there was lower variability in the anterior DMN (medial part of the superior frontal gyrus) in AD compared with aMCI (Supplementary Table 2).

ICA-based denoising method replicated the GSR-based finding that AD had lower variability than aMCI in the posterior DMN (angular gyrus) with the same FWE-corrected p < 0.05 cluster threshold, along with a smaller precuneus cluster at p < 0.05 uncorrected level (Fig. 2C, bottom panel, Supplementary Table 2). In contrast to the GSR results, there was higher SN variability (insula) in aMCI compared with HC (Fig. 2A, bottom panel). Furthermore, AD had lower variability in the posterior DMN (precuneus/angular gyrus) compared with HC (Fig. 2D, bottom panel), where aMCI displayed an intermediate level of DMN variability (precuneus) between AD and HC (Supplementary Fig. 1; HC > aMCI > AD).

Given that not only similar, but distinct slow4 results were found between GSR and ICA-based approach, we explored the possible explanations via examining the associations between the global signal time series and the voxel-level slow4 time series within each of the three groups and compared them using two-sample t-tests. We found that global signal presented differential associations with the DMN and SN time series at slow4 across groups (Supplementary results; Supplementary Fig. S2), which potentially explain the inconsistent results of slow4 using GSR and ICA approaches.

**Figure 3:**
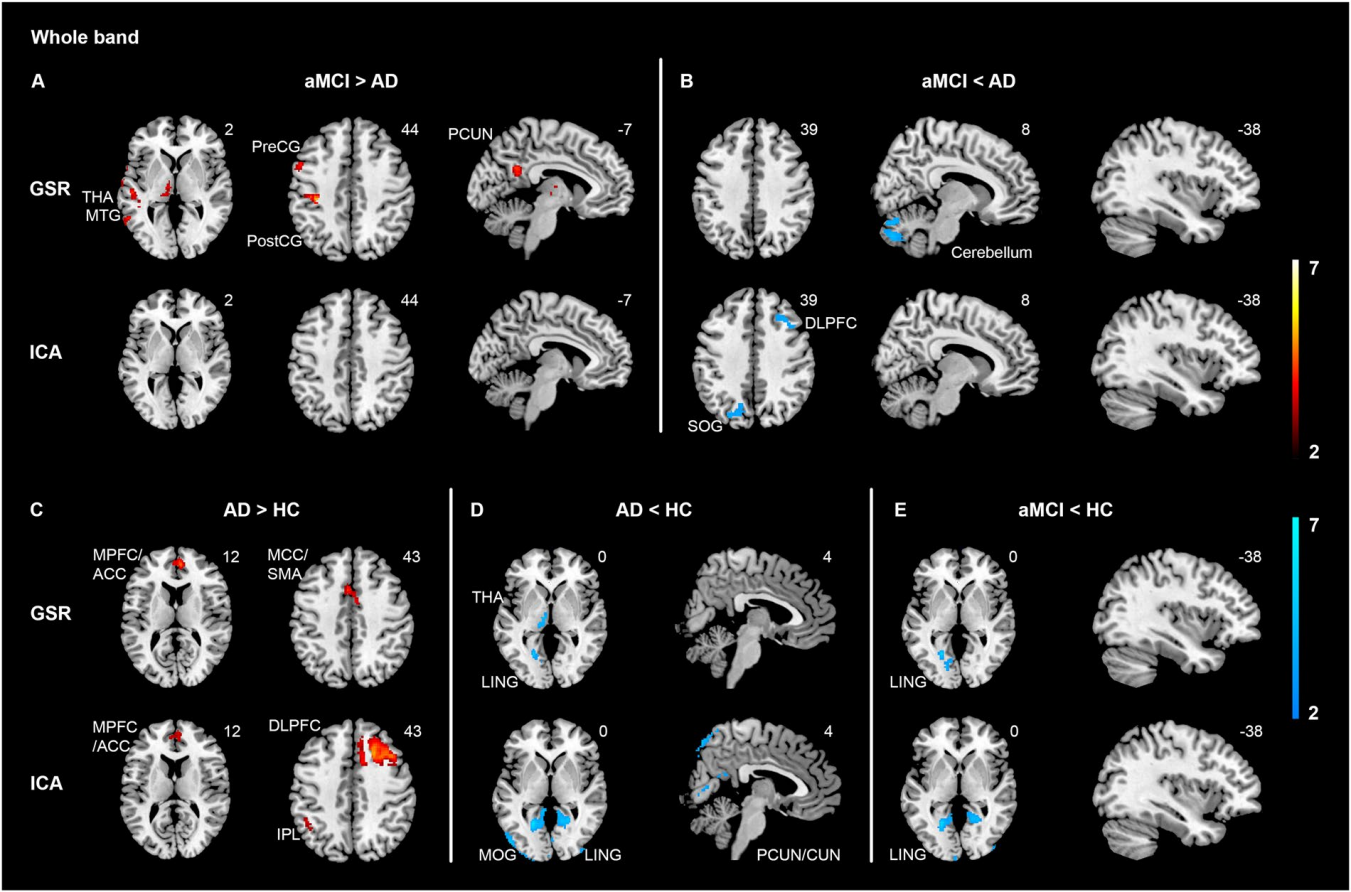
Whole band hemodynamic variability comparisons between AD, aMCI, and HC with GSR and ICA-based denoising. To ease results comparison between whole band and sub-frequency band (i.e., slow4 and slow5), same slices from results of slow5 (see Fig. 1) and slow4 (Fig. 2) were also displayed. Across contrasts, largely non-overlapping results were shown between whole band and slow4/slow5, using both GSR and ICA-based denoising methods. Specifically, using GSR approach, aMCI showed higher variability mainly in the DorsAttn and subcortical regions compared with AD (A, top panel), while lower variability in the cerebellum (B, top panel) and VN (E, top panel) compared with AD or HC. Moreover, AD showed increased variability in the anterior DMN (C, top panel) and reduced variability in the VN and subcortical regions (D, top panel) compared with HC. ICA-based data denoising replicated lower VN variability in aMCI compared with HC (E, bottom panel), higher variability in the anterior DMN (C, bottom panel) while lower VN variability (D, bottom panel) in AD compared with HC. Specific to data-denoising approach, ICA-based denoising revealed higher variability in the ECN (C, bottom panel) while lower variability in the posterior DMN/VN (D, bottom panel) in AD compared with HC. Results were obtained at p < 0.05 family wise error (FWE) correction on the cluster level, with a previous height threshold of p < 0.001, superimposing on the MNI brain template. We also reported results with a less stringent cluster-level threshold of p < 0.05 (uncorrected, k > 40), with a previous height threshold of p < 0.001 (for the PCUN at aMCI > AD with GSR [A, top panel] and the SOG and DLPFC at aMCI <AD [B, bottom panel] with ICA-based denoising). Colour bar represents T value. Abbreviations: ACC = Anterior cingulate cortex; AD = Alzheimer’s disease; aMCI = Amnestic mild cognitive impairment; CUN = Cuneus; DLPFC = Dorsolateral prefrontal cortex; DMN = Default mode network; DorsAttn = Dorsal attention network; ECN = Executive control network; GSR = Global signal regression; HC = Healthy controls; ICA = Independent component analysis; IPL = Inferior parietal lobule; LING = Lingual gyrus; MCC = Mid-cingulate cortex; MOG = Middle occipital gyrus; MPFC = Medial prefrontal cortex; MTG = Middle temporal gyrus; PCUN = Precuneus; PostCG = Postcentral gyrus; PreCG = Precentral gyrus; SMA = Supplementary motor area; SOG = Superior occipital gyrus; THA = Thalamus; VN = Visual network. (Color should be used for this figure in print; 2-column fitting image).

Moreover, we found distinct variability patterns of SD in the whole frequency band (Supplementary Table 3, Fig. 3), which did not overlap with those regions identified in slow4 and slow5. This indicated that the observed variability differences at slow4 and slow5 between groups were not due to differences of SD in the whole band.

In addition, controlling for motion and presence of significant cerebrovascular disease (CeVD) revealed comparable results at both slow5 (Supplementary Figs. S3 and S5) and slow4 (Supplementary Figs. S4 and S6).

### Correlation analyses of variability with baseline cognition, hippocampal volume and cognitive decline

**Figure 4:**
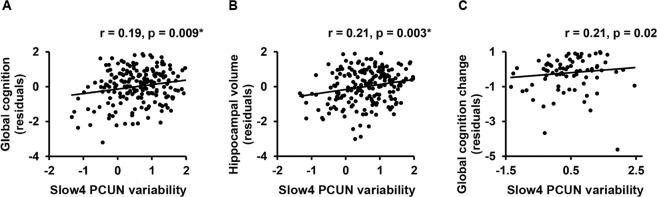
Slow4 DMN hemodynamic variability was associated with baseline global cognition, hippocampal volume and cognitive decline. After ICA-based denoising, lower variability in the posterior DMN was associated with poorer baseline global cognition (**A**) and smaller hippocampal volume (**B**) at slow4. Furthermore, over a 2-year follow-up, lower posterior DMN variability was associated with faster cognitive decline at slow4 in all patients (**C**). GSR approach showed similar correlation patterns as described in the text. ANG = angular gyrus; GSR = Global signal regression; ICA = Independent component PCUN = Precuneus. *Correlations surviving multiple comparisons correction. (2-column fitting image).

At slow4, lower variability in the posterior DMN was associated with worse global cognition and smaller hippocampal volume at baseline in all patients for both ICA-based denoising (Fig. 4A,B), and GSR approach (Supplementary Table 4).

At slow5, lower variability in the posterior DMN and higher variability in the SN were related to worse global cognition and smaller hippocampal volume respectively with GSR approach (Supplementary Table 3), which was absent after ICA-based denoising.

Regarding the correlation between variability and cognitive decline over a 2-year follow-up, lower variability in the posterior DMN at slow4 was associated with faster cognitive decline, which was consistent between the ICA-based denoising (Fig. 4C) and GSR approach (Supplementary Table 3).

## Discussion

BOLD signal variability is important for optimal brain functioning. To our best knowledge, our study is the first and the largest study so far to investigate the frequency-dependent patterns of resting state BOLD signal variability (SD) and their associations with cognitive impairment/decline in AD and aMCI, repeating with two separate data denoising approaches. Briefly, we found that both GSR and ICA-based denoising approaches converged to show a reciprocal balance of frequency-specific variability changes in the DMN and SN in aMCI compared with AD and/or HC. Importantly, for both data denoising methods, lower posterior DMN variability at slow4 related to poorer global cognition and smaller hippocampus at baseline, and faster cognitive decline over 2-year follow-up. Our findings on divergent DMN-SN frequency-specific variability changes may represent an important mechanism underlying brain functioning deterioration in early AD.

Consistent with previous findings of disrupted DMN network in aMCI^10–12^, altered DMN variability was observed in the present study. We found increased DMN variability in aMCI with GSR at both slow4 and slow5, while a recent meta-analysis of variability in aMCI reported decreased DMN variability^28^. This discrepancy may be explained by several differences, including the differences in sample size (103 aMCI in the present study vs. 26 aMCI on average in the meta-analysis study), methods of variability calculation (SD of the BOLD signal in the present study vs. ALFF in the meta-analysis study) and whether different frequency bands were tested separately etc. “Further studies are needed to refine factors” that could better account for the complex variability pattern at different frequency bands in aMCI. The DMN has been suggested to be associated with internal processing of self at rest, such as self-reflection, retrieving memory, or thinking of one’s future^33^. Therefore, it might be speculated that the DMN is a key network showing early disruption in aMCI in terms of aberrant hyper-activity, affecting self-related processing in aMCI.

In contrast, we found lower SN variability in aMCI compared with AD and/or HC at both slow4 and slow5 with GSR, which is in line with previous variability studies in aMCI with GSR, ICA or neither^28^. With the insula as a key node, it has been suggested that the SN plays an important role in salience processing, including detecting salient information and directing attention toward or away from internal processing in concert with the DMN^34^. It would be interesting for future studies to combine resting-state and task-based fMRI data to test whether the observed lower SN variability and increased DMN variability in aMCI (compared with AD/HC) are associated with each other and how these network dynamics contribute to attention, memory and self-related processing in aMCI.

Importantly, we replicated the observed DMN-SN balance at slow5 via a separate ICA-based denoising. However, we observed a tendency of DMN-SN balance at slow4 in the opposite direction following ICA-based denoising. Specifically, although direct comparison between aMCI and HC did not show significant group difference at slow4 (Supplementary Table 2), we found that ICA-denoising revealed lower DMN variability (cluster from the comparison between HC and AD, Supplementary Fig. S1), and higher SN variability in aMCI compared with HC. Nonetheless, both GSR and ICA results suggest a divergent variability changes between the DMN and SN.

There was lower variability in the posterior DMN in AD compared with HC at both slow4 and slow5 only after ICA-based denoising, replicating some previous findings^24,26^, but not others^25,27^. Most of the previous studies in AD did not perform GSR or ICA-based denoising. Only one study used primary component analysis to regress out signals of no interest and found no difference between AD and HC, but suffered from small sample size of AD patients (n = 10)^27^.

It should be noted that currently there is no gold standard on fMRI pre-processing methods, and previous evidence has indeed shown that whether use GSR or other denoising methods could result in different results^35^. Our discrepant results in AD compared with HC between GSR approach and ICA-based denoising can be possibly explained by the weaker associations of global signal with the DMN time series in AD compared with HC (Supplementary Fig. S2A), and therefore regressing out global signal (GSR approach) may result in reduced group differences in variability between AD and HC. Similarly, we speculate that the opposite SN variability between the two data pre-processing methods at slow4 in aMCI compared with HC was due to a stronger association between global signal and SN time series in aMCI (Supplementary Fig. S2B). Nevertheless, both approaches converge to suggest a dynamical DMN-SN balance. Notably, anti-correlated pattern of functional connectivity between the DMN and SN has been found in AD and behavioural variant frontotemporal dementia^7,8^. The divergent variability patterns between the DMN and SN in aMCI as observed in the present study further support the reciprocal relationships between these two networks. This may suggest that DMN-SN balance in variability plays an important role at the prodromal AD stage.

Our findings of divergent DMN-SN variability changes at aMCI stage may reflect compensatory processes. Briefly, segregation and integration balance between brain areas has been proposed to achieve optimal brain functioning^15^. Indeed, brain networks balance has been found at initial healthy ageing stage, with reduced resting-state functional connectivity within brain networks (reduced integration) and decreased functional connectivity between brain networks (increased segregation), but then followed by reductions in both as ageing proceeded^36^. The authors suggested that the initial balance between brain networks segregation and integration represented a compensatory effort during ageing process. In line with the balance perspective, we found balanced/divergent DMN-SN variability patterns in aMCI compared with AD/HC. Importantly, following both GSR and ICA-based denoising, the lower posterior DMN variability at slow4 was not only associated with poorer cognition and smaller hippocampal volume at baseline, but also faster cognitive decline. For GSR only, reduced posterior DMN variability at slow5 was also associated with worse baseline global cognition, and smaller hippocampal volume was related to higher SN variability. We speculated that the reciprocal balance between the DMN and SN in aMCI may represent an effort to obtain balance between networks, serving as a compensatory mechanism to avoid further cognitive deterioration. Indeed, compensational mechanisms have been found in aMCI, such as enhanced functional connectivity, effective connectivity between networks, or increased activity compared with controls^37–39^.

Taken together, the observed divergent DMN-SN variability pattern in aMCI may play an essential role in gaining balance between networks to maintain cognitive functioning. This may represent a compensational pattern against cognitive decline and disease progression in aMCI. Because of the close link between variability and other brain measures such as functional connectivity^19,40^, multimodal studies are encouraged to elaborate how BOLD variability interact with other brain structural and functional abnormalities or lead to downstream neurodegeneration in early AD and how to intervene to allow aMCI individuals to maintain cognitive ability and possibly slow down disease progression.

One important strength of this study was assessing variability separately at slow4 and slow5. Frequency-dependent variability alterations in region-specific brain areas have been reported at slow4 and slow5 in different disorders^41–46^. These results suggest both disease- and frequency-dependent disruptions of variability patterns. There has only been one study that investigates frequency-dependent variability in aMCI patients, using ALFF and fALFF^31^. The authors demonstrated an interaction of variability between frequency bands (slow4 and slow5) and group (aMCI vs. HC) in the angular gyrus and small clusters in the occipital and parietal lobule, which was due to group differences at slow5 only. However, this study suffered from small sample size (n = 24), lack of GSR/ICA-based denoising, and did not find any variability-cognition associations.

Differently, using BOLD signal SD, we found both overlapping and distinct patterns of variability changes between slow4 and slow5 in aMCI. This is in line with previous findings that there are both frequency-general (e.g., presence of typical resting-state networks such as the DMN)^47^ and frequency-specific features (e.g., spatial extent, homogeneity, variability and functional connectivity strength)^48^ across slow4 and slow5. Taken together with the common and unique correlation patterns of variability with cognition/cognitive decline and hippocampal volume between slow4 and slow5, we propose that slow4 and slow5 may have both mutual and differential contributions to the cognitive profile and network-specific neurodegeneration along the AD spectrum. Moreover, comparing results between GSR and ICA-based denoising showed more overlapping patterns for slow5 than slow4. This might imply that slow4 (higher frequency) is more vulnerable to data pre-processing approaches. Notably, our exploration in the whole band showed largely non-overlapping variability patterns in comparison with slow4 and slow5 (Supplementary Table 3, Fig. 3), necessitating investigation into sub-frequency bands. Future longitudinal studies are encouraged to further elaborate the effects of different frequency bands and different data pre-processing approaches including more advanced approaches to remove physiological noise^49^, which may provide complimentary information.

The present study had some limitations. Firstly, our study was cross-sectional. Longitudinal studies would be interesting to track the variability changes along disease progression by comparing with their normal trajectories across the human life span^50,51^. Secondly, how frequency and network specific variability relates to disease markers (e.g., amyloid and tau) remain unclear. Future multimodal neuroimaging methods would be of help to test potential interaction effects between variability and disease markers. Finally, it would be interesting to investigate whether and how regional BOLD variability patterns relate to functional connectivity between regions and their possible joint contribution to disease deterioration.

## Conclusion

To conclude, we observed reciprocal DMN-SN variability balance in aMCI compared with AD/HC, possibly representing functional compensation in aMCI before brain functional network breakdown and clinical progression to AD. Further support stemmed from the correlation findings that lower posterior DMN variability at slow4 was associated with poorer cognition and smaller hippocampal volume at baseline, and predicted faster cognitive decline over time in cognitively impaired patients (i.e., AD and aMCI combined). Our findings showed that slow4 and slow5 BOLD variability presented both overlapping and differential patterns of spatial changes and correlations with hippocampal volume and cognition in AD spectrum. Moreover, despite of the converging findings between GSR and ICA approaches, we found discrepancy between the two methods especially at slow4, which might be due to the differential associations between global signal and BOLD signals in the DMN and SN regions across groups. The present findings underscore the importance of frequency-specific investigation of BOLD variability, and might facilitate future intervention design in early AD based on the relationship between DMN/SN network breakdown and cognitive decline.

## Methods

### Participants

We studied 124 AD, 103 aMCI, and 49 HC from an ongoing project, recruited from memory clinics in the National University Hospital, Saint Luke’s Hospital and nearby communities^52,53^. Diagnoses were made by psychologists, neurologists, and research personnel at weekly consensus meetings based on clinical observation, lab tests (e.g., blood test), neuroimaging scans and neuropsychological assessments. Accordingly, participants fulfilling the criteria of National Institute of Neurological and Communicative Diseases and Stroke-Alzheimer’s Disease and Related Disorders Association (NINCDS-ADRDA)^54^ were identified as AD. aMCI patients were identified if participants had both subjective cognitive complaint and objective impairment in at least the memory domain (see *Clinical and neuropsychological assessments*), but not demented and remained functionally independent. Finally, HC were also included who showed no objective cognitive impairment based on the neuropsychological assessments, scored ≥ 26 on the Mini–Mental State Examination (MMSE) (see *Clinical and neuropsychological assessments*), and had no significant CeVD (see *Supplement* for definition of significant CeVD). After image QC (see *data pre-processing*), 28 AD, 5 aMCI and one HC were excluded. In summary, 96 AD, 98 aMCI and 48 HC were included in the final analyses. The excluded AD patients were older and more severely impaired compared with the included AD, without group differences in sex, handedness, ethnicity and education years (Supplementary Table 5). Participants’ demographic and neuropsychological assessment performance (clinical scores and global cognition) are described in Table 1 (see *Supplement* for participants inclusion/exclusion criteria).

**Table 1:** Demographic and neuropsychological features of participants.

	HC (n = 48)	aMCI (n = 98)	AD (n = 96)	F/χ^2^	*p*^a^
Age, yrs	72.04 (4.07)^e^	72.39 (7.24)^e^	74.43 (7.13)	3.04	0.05
Male/Female	21/27	52/46	37/59	4.19	0.12
Handedness, R/L	45/3	95/3	94/2	1.77	0.41
Ethnicity, C/non-C	43/5	81/17	74/22	3.44	0.18
Education, yrs	10.08 (4.78)	6.88 (4.91)^c^	4.92 (4.93)^c,d^	17.93	<0.001*
Global cognition^b^	0.00 (1.00)	−3.29 (2.41)^c^	−7.47 (3.13)^c,d^	151.27	<0.001*
Global cognitive decline^b^	−0.07 (0.48)	−0.05 (1.20)	−1.81 (2.20)^c,d^	23.20	<0.001*
CDR-SOB	0.14 (0.35)^d,e^	0.90 (0.90)^e^	6.70 (2.73)	321.85	<0.001*
MMSE	27.46 (1.90)	23.91 (3.74)^c^	16.10 (4.40)^c,d^	179.85	<0.001*
MoCA	24.38 (2.50)	19.15 (4.66)^c^	11.13 (4.67)^c,d^	170.51	<0.001*
CeVD status, Y/N	0/48	52/46^c^	46/50^c^	41.28	<0.001*
Ischemic heart disease, Y/N	2/46	9/89	8/88	1.17	0.56
Hypertension, Y/N	26/22	68/30	74/22^c^	7.92	0.019*

Values represent mean (s.d). Groups were compared on the listed variables with ANOVAs or chi-square tests where appropriate, with a threshold of *p* < 0.05 (two-tailed, *). ^a^*p* values from the ANOVA between all three groups. ^b^Global cognition value represents standardized z-score of global cognition (one AD did not have global cognition data), and global cognitive decline was defined as the difference between baseline and year 2 (year 2 minus baseline; longitudinal cognition was not available for 43 AD, 25 aMCI and 16 HC). Significance of post-hoc pairwise comparisons (*p* < 0.05) was indicated if group mean was lower compared with or distribution different from HC (c), MCI (d) or AD (e). AD = Alzheimer’s disease; aMCI = Amnestic mild cognitive impairment; C/non-C = Chinese/non-Chinese; CDR-Global = Clinical Dementia Rating Scale Global Score; CDR-SOB = Clinical Dementia Rating Scale Sum of Boxes; CeVD = Cerebrovascular disease; HC = Healthy controls; MMSE = Mini-Mental State Examination; MoCA = Montreal Cognitive Assessment; R/L = Right/left; Y/N = Yes/No.

The study was approved by the SingHealth Institutional Review Board and the National Healthcare Group Domain-Specific Review Board, in accordance with the Declaration of Helsinki. Written informed consents were provided by all participants.

### Clinical and neuropsychological assessments

The Montreal Cognitive Assessment, MMSE, Clinical Dementia Rating and a locally validated neuropsychological assessment battery were administered to all participants by trained psychologists or clinicians^55^. The assessment battery consists of tests assessing two memory domains, namely verbal memory and visual memory, and five non-memory domains, namely executive function, attention, language, visuomotor speed and visuoconstruction. A standardized global cognition z score was obtained following previous publication^52^, with higher z score indicated better cognition.

### Image acquisition

The fMRI scanning was performed in a 3 T Siemens Magnetom Tim Trio scanner using a 32-channel head coil at Clinical Imaging Research Centre, National University of Singapore. A whole-brain T1-weighted anatomical image was acquired, using magnetization prepared rapid gradient recalled echo (MPRAGE) sequence (192 sagittal slices, TR = 2300 ms, TE = 1.9 ms, TI = 900 ms, flip angle = 9°, FOV = 256 × 256 mm^2^, slice thickness = 1 mm, voxel size = 1 × 1 × 1 mm^3^). For the T2*-weighted resting-state functional scanning, data were collected in the axial plane with an interleaved collection with participants’ eyes closed (48 slices, duration = 5.01 min, TR = 2300 ms, TE = 25 ms, flip angle = 90°, FOV = 192 × 192 mm^2^, slice thickness = 3 mm, voxel size = 3 × 3 × 3 mm^3^).

### Data analysis

#### Data pre-processing

fMRI data were pre-processed with a standard pipeline using the FMRIB Software Library (FSL)^56^ and Analysis of Functional NeuroImages software (AFNI)^57^ as described previously^36,53^. Briefly, pre-processing of the structural images included reducing nonlinear image noise (SUSAN), extracting brain tissue (skull stripping, BET), normalizing to the Montreal Neurological Institute (MNI) 152 standard space (FLIRT/FNIRT) and segmenting into grey matter (GM), white matter (WM) and cerebrospinal fluid (CSF). Pre-processing steps for the functional resting-state data included excluding the first five volumes for magnetic field stabilization, motion correction, despiking and grand-mean scaling, spatial smoothing with a 3D 6-mm full-width/half-maximum (FWHM) Gaussian kernel, temporal band-pass filtering (whole band: 0–0.25 Hz; standard low frequency range: 0.009–0.1 Hz) and detrending, co-registering to the anatomical image (BBR) and subsequently to the MNI 152 standard space (FNIRT), and finally regressing out nuisance signals from CSF, WM, whole-brain global signal and six motion parameters. Additionally, two separate sub-bands were extracted from the standard low frequency range (0.009–0.1 Hz) following previous literature^41,58^, including slow4 (0.027–0.073 Hz) and slow5 (0.01–0.027 Hz). Participants with excessive head motion (maximum absolute motion >4 mm) and poor image quality (e.g., incomplete scan, failed pre-processing QC) were excluded. There were larger head motion (maximum absolute) in AD (mean ± s.d. = 1.75 ± 1.25) compared with aMCI (mean ± s.d. = 1.38 ± 1.07) and HC (mean ± s.d. = 1.24 ± 1.15) (ps < 0.05), without statistically significant difference between the latter two (p = 0.50). We therefore further controlled for motion in our statistical analyses.

Moreover, due to the controversy over GSR^35,59^ (e.g., whether it induces spurious anti-correlations between regions, whether it regresses out not only noise but also signal), we also applied another pre-processing pipeline for the functional resting-state data using ICA-based denoising, which was commonly used in previous studies of BOLD variability^17,60^. Briefly, (1) the same standard pre-processing steps as the GSR approach were first performed, including excluding the first five volumes, correcting for motion, spatial smoothing with a 3D 6-mm FWHM Gaussian kernel, grand-mean scaling, co-registering to the anatomical image (BBR) and subsequently to the MNI 152 standard space (FNIRT). (2) Single-session ICA was conducted per participant to decompose data into independent components, with a high pass filter cut-off of 0.01 Hz and automatic dimensionality estimation (FSL/MELODIC)^61^. (3) FSL-FIX^62,63^ was applied to automatically identify ICA components as noise or signal, using our data-specific trained-weights (see next paragraph for details). 4) Finally, the identified noise components were removed from the resting-state data to obtain the denoised data for subsequent analyses.

Regarding the study-specific training, we randomly selected 10 participants from each of the three groups (n = 30 in total) as the training subjects. For each participant, the resulting ICA components were manually classified into signal/noise as agreed between two raters (L.Z. and K.K.N) following criteria described in previous work^64^, which included (1) motion-related components (e.g., ring effect, sudden time series spikes or low frequency signal drift), (2) vein-related components (e.g., signal from the sagittal sinus), (3) components in relation to non-grey matter (e.g, cerebrospinal fluid, white matter), 4) components with high frequency and high power, and 5) MRI-related components and components with sparsity (i.e., non-discernible spatial pattern alternating between negative and positive values). We used relatively conservative rejection criteria to keep signals of interest as much as possible as suggested previously^64^. This resulted in the highest balance ratio of 89.3% between true-positive rate (TPR, rate of identifying signal components correctly) and true-negative rate (TNR, rate of identifying noise components correctly) at the threshold of 20, following the recommended formula: (3*TPR + TNR)/4^62,63^.

#### Variability analysis of resting-state data

Resting-state fMRI data were analysed using the Statistical Parametric Mapping (SPM12, v.6470, www.fil.ion.ucl.ac.uk/spm) and Matlab 7.11.0 (R2010b; the Math Works Inc., Natick, MA). We focused on slow4 and slow5 because these two bands have been suggested to have functional meanings instead of random noise or nuisance signals from white matter activity or physiological processes (e.g., respiration)^2,65^. An index of variability was calculated based on a method reported previously^21^. Briefly, at each voxel, the SD of the BOLD signal was first calculated in the whole band (0–0.25 Hz) and sub-bands, i.e., slow4 (0.027–0.073 Hz) and slow5 (0.01–0.027 Hz), separately. Fractional SD (fSD) in each sub-band was then obtained by dividing the SD of the sub-band by the SD in the whole band, representing sub-band specific contribution relative to the total BOLD signal variability. Finally, voxel-wise fSD maps were converted into z-score maps (z-fSD) by standardizing fSDs spatially across the whole brain in slow4 and slow5 separately, resulting in two z-fSD maps for each participant.

To examine whole-brain group differences in variability between HC, aMCI and AD, z-fSD maps at slow4 and slow5 were entered into separate one-way ANCOVAs, with group as the independent variable, and age, sex, education and total grey matter volume (GMV; see *supplementary methods*) as covariates of no interest. Main effect of group was tested, followed by pair-wise comparisons between any of the two groups.

To exclude potential confounding effects from difference in the whole band, a one-way ANCOVA was conducted to compare z-score maps of SD in the whole band between AD, aMCI and HC, controlling for age, sex, education, and GMV. Further validations have been done by taking maximum absolute motion or presence of significant CeVD as additional nuisance variables.

For all analyses, statistical threshold was set at a voxel-defining threshold of p < 0.001, followed by a p < 0.05 FWE corrected at the cluster level. Moreover, to facilitate comparison between the GSR approach and ICA-based denoising, we also reported results at a lower threshold surviving a voxel-level threshold of p < 0.001 and a cluster-level threshold of p < 0.05 (uncorrected) and k ≥ 40.

#### Correlation analysis

We performed correlation analyses of the variability with 1) global cognition at baseline; and 2) bilateral hippocampal volume at baseline (see *supplementary methods*).

Furthermore, we examined whether the variability related to global cognition decline over time, defined as the difference between baseline and year 2 (year 2 minus baseline).

For all correlation analyses, we focused on the brain clusters showing group differences between aMCI and HC or between AD and HC (including clusters surviving the voxel-level threshold consistently using both GSR and ICA-based denoising) (n = 3 for slow4, and n = 6 for slow5 for both data denoising methods). Correlation analyses were performed in all patients after controlling for age, sex, education years and total GMV. The correlation analyses were performed within each frequency band separately, applying Bonferroni correction for the number of clusters of interest per frequency band. Variability estimates of the clusters were extracted using MarsBaR (http://marsbar.sourceforge.net). Statistical significance was set at p < 0.05 (two-tailed).

## Supplementary information

Supplementary material.
