# Supplementary material for: Distinct BOLD variability changes in the default mode and salience networks in Alzheimer’s disease spectrum and associations with cognitive decline
Source: Sci Rep. 2020 Apr 15;10:6457. doi: 10.1038/s41598-020-63540-4 (PMC7160203; doi:10.1038/s41598-020-63540-4)
Supplement: Supplementary file 1 — Supplementary material. [file 41598_2020_63540_MOESM1_ESM.doc]

# Distinct BOLD variability changes in the default mode and salience networks in Alzheimer’s disease spectrum and associations with cognitive decline

­­

**Authors:** Liwen Zhang, Xi-Nian Zuo, Kwun Kei Ng, Joanna Su Xian Chong, Hee Youn Shim, Marcus Qin Wen Ong, Yng Miin Loke, Boon Linn Choo, Eddie Jun Yi Chong, Zi Xuen Wong, Saima Hilal, Narayanaswamy Venketasubramanian, Boon Yeow Tan,

Christopher Li-Hsian Chen, Juan Helen Zhou^*^

*** Corresponding author**

Juan Helen Zhou, Ph.D.

Address: Tahir Foundation Building (MD1), 12 Science Drive 2, #13-05C, National University of Singapore, Singapore 117549

Tel: (65) 66012392 | Fax: (65) 62218685

Email: helen.zhou@nus.edu.sg

**SUPPLEMENTARY MATERIALS**

**1 SUPPLEMENTARY METHODS**

**1.1 Exclusion criteria of participants**

**1.2 Voxel-based morphometry (VBM)**

**2 SUPPLEMENTARY RESULTS**

**2.1 Correlation analyses of global signal with slow4 time series**

**Supplementary Table 1** Slow5 BOLD variability comparisons between AD, aMCI, and HC with GSR or ICA-based denoising

**Supplementary Table 2** Slow4 BOLD variability comparisons between AD, aMCI, and HC with GSR or ICA-based denoising

**Supplementary Table 3** Whole band BOLD SD (z-score) comparisons between AD, aMCI, and HC with GSR or ICA-based denoising

**Supplementary Table 4** Correlations between BOLD variability and baseline global cognition, hippocampal volume and global cognitive decline over a 2-year follow-up in all patients using both GSR or ICA-based denoising

**Supplementary Table 5** Group comparisons of demographic and neuropsychological features between included and excluded AD.

**Supplementary Figure S1** Lower DMN variability in AD and aMCI compared with HC at slow4 with ICA-based denoising

**Supplementary Figure S2** Divergent correlations of global signal with slow4 time series in the default mode network and salience network in AD compared with HC

**Supplementary Figure S3** Divergent slow5 hemodynamic variability changes in the default mode and the salience networks in amnestic MCI and AD controlling for motion

**Supplementary Figure S4** Divergent slow4 hemodynamic variability changes in the default mode and the salience networks in amnestic MCI and AD controlling for motion

**Supplementary Figure S5** Divergent slow5 hemodynamic variability changes in the default mode and the salience networks in amnestic MCI and AD controlling for cerebrovascular disease **Supplementary Figure S6** Divergent slow4 hemodynamic variability changes in the default mode and the salience networks in amnestic MCI and AD controlling for cerebrovascular disease

**1 SUPPLEMENTARY METHODS**

**1.1 Exclusion criteria of participants**

Participants were excluded if they had any of the following: 1) comorbidity with bipolar disorder or schizophrenia; 2) epilepsy that was hard to control and might lead to cognitive impairments; 3) space occupying intracranial mass lesion (e.g., tumor); 4) normal or obstructive pressure hydrocephalus; 5) hypertensive, hypotensive, anoxic/hypoxic, uremic or hepatic encephalopathy; 6) nutritional, traumatic or toxic disorder that could affect the central nervous system; 7) current or a history of substance abuse disorder as defined by the Diagnostic and Statistical Manual of Mental Disorders (DSM-IV) that may have an influence on the central nervous system (e.g., alcohol, phencyclidine, opiates, barbiturates or cocaine); 8) infections of the central nervous system such as viral encephalitis, bacterium, syphilis, Creutzfield-Jacob disease, fungi, rickettsiae, or tuberculosis; 9) intracerebral hemorrhage that could lead to cognitive impairment; 10) moyamoya disease, cranial arteritis or central nervous system inflammatory vasculitides; and 11) MRI-incompatibilities (e.g., metal implants, pregnancy). Healthy controls were free of significant cerebrovascular disease (CeVD) that was defined as ≥ 2 lacunes, and/or ≥ 1 cortical infarct, and/or ≥ 8 on the Age Related White Matter Changes Scale following previous publication^1^.

**1.2 Voxel-based morphometry (VBM)**

Given its involvement as an marker of Alzheimer’s disease (AD)^2,3^, hippocampal volume was calculated for each participant, using VBM8 toolbox (<http://www.neuro.uni-jena.de/vbm/download/>) embedded in SPM12 (v.6470, [www.fil.ion.ucl.ac.uk/spm](http://www.fil.ion.ucl.ac.uk/spm)). Briefly, the structural images were first segmented into grey matter (GM), white matter (WM) and cerebrospinal fluid, using the adaptive Maximum A posterior technique^4^. After that, a data-specific template was created using the DARTEL tool based on previous GM and WM segments. GM and WM probability maps were then registered to the created DARTEL template in MNI standard space. Finally, modulation was applied to account for individual brain volumes, multiplying the voxel values by the non-linear component of the Jacobian determinant.

To obtain GM volume in the hippocampus, a binary mask of bilateral hippocampus was created based on the Automated Anatomical Labelling labels (AAL)^5^ in the WFU Pick Atlas toolbox. Moreover, whole-brain GM was also calculated to be used as a covariate in the analyses.

# 2 SUPPLEMENTARY RESULTS

**2.1 Correlation analyses of global signal with slow4 time series**

# We found that AD had a stronger association of global signal with the time series in the salience network (SN) and a weaker one with the time series in the posterior default mode network (DMN) at slow4 compared with HC, with aMCI displaying an intermediate level between AD and HC (Supplementary Fig. S2). Because of such group differences, we speculate that the impact of GSR are partly less reductions of DMN slow4 variability in AD and aMCI compared to HC, resulting in null DMN findings in AD < HC contrast (Fig. 2D) and greater DMN in aMCI > HC contrast (Fig. 2A). In parallel, GSR leads to more reductions of SN slow4 variability in AD and aMCI compared to HC, resulting in the SN difference in the contrast of aMCI < HC (Fig. 2B).

# Supplementary Table 1 Slow5 BOLD variability comparisons between AD, aMCI, and HC with GSR or ICA-based denoising.

| **GSR** | | | | | | | **ICA** | | | | | | |
| --- | --- | --- | --- | --- | --- | --- | --- | --- | --- | --- | --- | --- | --- |
| **Regions** | **k** | **BA** | **Z** | **MNI coordinates** | | | **Regions** | **k** | **BA** | **Z** | **MNI coordinates** | | |
|  |  |  |  | x | y | z |  |  |  |  | x | y | z |
| **Main effect of group** |  |  |  |  |  |  |  |  |  |  |  |  |  |
| L HIP/AMYG | 61 |  | 4.73* | -28 | -14 | -10 |  |  |  |  |  |  |  |
| L ANG | 96 | 39/40 | 4.69* | -58 | -60 | 30 | L ANG | 42 |  | 4.34 | -44 | -60 | 32 |
| L INS/ROL | 122 |  | 4.70* | -36 | 4 | 0 | L INS/ROL | 55 | 13 | 4.69 | -38 | -4 | 14 |
| R PUT | 109 |  | 4.87* | 24 | 8 | 4 |  |  |  |  |  |  |  |
| L PHG | 68 |  | 4.17* | -16 | -26 | -20 |  |  |  |  |  |  |  |
|  |  |  |  |  |  |  | R CUN | 169 | 7/19 | 4.70* | 2 | -80 | 34 |
|  |  |  |  |  |  |  | L/R PCUN/PCC | 201 | 23/30/31 | 4.29* | -4 | -52 | 22 |
|  |  |  |  |  |  |  | R PCUN | 82 | 7 | 3.95 | 8 | -54 | 60 |
|  |  |  |  |  |  |  | L PCUN | 68 | 7 | 4.14 | -4 | -66 | 46 |
| L ACC | 41 | 32 | 5.45 | -12 | 36 | 14 |  |  |  |  |  |  |  |
| L LING/FFG | 88 | 18/19 | 5.47 | -22 | -82 | -16 |  |  |  |  |  |  |  |
| L PostCG | 40 |  | 5.41 | -36 | -14 | 40 |  |  |  |  |  |  |  |
| L PostCG | 65 | 40 | 4.20 | -48 | -44 | 60 |  |  |  |  |  |  |  |
|  |  |  |  |  |  |  | R PostCG | 52 |  | 4.00 | 56 | -8 | 22 |
| R SPL | 85 | 7 | 4.26 | 28 | -60 | 60 |  |  |  |  |  |  |  |
|  |  |  |  |  |  |  | L SPL | 52 |  | 3.99 | -32 | -48 | 58 |
| R STG/INS | 44 |  | 4.12 | 38 | -12 | -4 |  |  |  |  |  |  |  |
|  |  |  |  |  |  |  | L SMG | 46 | 40 | 4.90 | -48 | -32 | 26 |
|  |  |  |  |  |  |  | L MTG | 53 |  | 4.46 | -54 | -66 | 14 |
| L Cerebellum | 42 |  | 4.64 | -6 | -60 | -12 |  |  |  |  |  |  |  |
| **aMCI > AD** |  |  |  |  |  |  |  |  |  |  |  |  |  |
| L LING/FFG | 140 | 18/19 | 5.49* | -22 | -82 | -16 |  |  |  |  |  |  |  |
| L ANG | 162 | 39/40 | 4.88* | -58 | -60 | 30 |  |  |  |  |  |  |  |
| L/R PCUN/ CUN/ SOG/ MOG/ IOG | 3733 | 7/18/19 | 5.36* | 2 | -78 | 36 | L/R PCUN | 197 | 7 | 4.68 | -4 | -66 | 46 |
|  |  |  |  |  |  |  | L/R PCUN | 294 | 7 | 4.67* | 4 | -56 | 20 |
|  |  |  |  |  |  |  | L PCUN | 80 | 7 | 3.82 | -10 | -74 | 40 |
|  |  |  |  |  |  |  | L MTG | 161 |  | 4.53* | -54 | -66 | 14 |
| L Cerebellum | 333 |  | 6.84* | -14 | -75 | -52 |  |  |  |  |  |  |  |
| R Cerebellum | 362 |  | 6.05* | 28 | -78 | -52 | R Cerebellum | 212 |  | 4.36* | 30 | -74 | -18 |
| L PostCG/IPL | 130 | 40 | 4.61 | -48 | -44 | 60 |  |  |  |  |  |  |  |
| R SPL | 141 | 7 | 4.44 | 28 | -60 | 60 |  |  |  |  |  |  |  |
| R CUN | 70 | 7 | 3.86 | 16 | -76 | 42 |  |  |  |  |  |  |  |
|  |  |  |  |  |  |  | L SPL | 94 | 7 | 4.03 | -26 | -68 | 42 |
| L Cerebellum | 50 |  | 4.09 | -18 | -92 | -30 |  |  |  |  |  |  |  |
| **aMCI < AD** |  |  |  |  |  |  |  |  |  |  |  |  |  |
| L AMYG/HIP | 76 |  | 5.11* | -24 | -6 | -16 | / |  |  |  |  |  |  |
| R PUT | 148 |  | 4.63* | 24 | 0 | 10 |  |  |  |  |  |  |  |
| L ROL/ INS | 88 |  | 4.19* | -34 | -4 | 18 |  |  |  |  |  |  |  |
| R INS/PUT | 101 |  | 4.09* | 36 | -14 | -4 |  |  |  |  |  |  |  |
| L ACC | 62 | 32 | 5.59 | -12 | 36 | 14 |  |  |  |  |  |  |  |
| R PARC | 50 |  | 6.37 | 2 | -46 | 74 |  |  |  |  |  |  |  |
| L ACC | 62 | 32 | 5.59 | -12 | 36 | 14 |  |  |  |  |  |  |  |
| L ACC/MCC | 46 | 24 | 5.05 | -4 | 10 | 28 |  |  |  |  |  |  |  |
| R SMG | 68 |  | 5.25 | 48 | -38 | 28 |  |  |  |  |  |  |  |
| L PostCG | 51 |  | 5.00 | -38 | -14 | 38 |  |  |  |  |  |  |  |
| R SMA | 40 |  | 4.95 | 12 | 12 | 50 |  |  |  |  |  |  |  |
| L PreCG | 48 | 6 | 4.86 | -34 | 2 | 42 |  |  |  |  |  |  |  |
| L PAL/ PUT | 46 |  | 4.45 | -10 | 2 | -6 |  |  |  |  |  |  |  |
| **aMCI < HC** |  |  |  |  |  |  |  |  |  |  |  |  |  |
| L INS/ ROL | 175 | 13 | 5.10* | -36 | 4 | 0 | L INS/ROL | 68 | 13 | 4.98 | -40 | -2 | 14 |
| R INS/ IFGoperc | 99 | 13 | 4.46* | 44 | 8 | 4 |  |  |  |  |  |  |  |
| R PUT | 202 |  | 5.07* | 24 | 10 | 4 |  |  |  |  |  |  |  |
| L PHG | 134 |  | 4.30* | -16 | -26 | -20 |  |  |  |  |  |  |  |
| L Cerebellum | 77 |  | 4.91* | -8 | -60 | -12 |  |  |  |  |  |  |  |
| L ACC | 69 | 24/32 | 4.24 | -12 | 36 | 14 |  |  |  |  |  |  |  |
| L PUT/HIP | 60 |  | 4.54 | -28 | -14 | -10 |  |  |  |  |  |  |  |
| L PUT | 40 |  | 4.92 | -22 | 0 | 10 |  |  |  |  |  |  |  |
| R INS | 45 | 13 | 4.84 | 34 | -26 | 10 |  |  |  |  |  |  |  |
| L STG | 59 |  | 4.27 | -46 | -38 | 10 |  |  |  |  |  |  |  |
|  |  |  |  |  |  |  | R PostCG | 88 |  | 4.33 | 52 | -12 | 16 |
|  |  |  |  |  |  |  | L SMG | 66 | 40 | 5.30 | -48 | -30 | 26 |
|  |  |  |  |  |  |  | L SPL | 68 |  | 4.35 | -32 | -48 | 58 |
| **aMCI > HC** |  |  |  |  |  |  |  |  |  |  |  |  |  |
| L/R PCUN/ANG/CUN  /MOG/ SOG | 5577 | 7/17/18/19/39 | 6.27* | -26 | -96 | 14 | L ANG | 156 | 39 | 4.55* | -44 | -60 | 32 |
| L LING | 44 | 18 | 4.49 | -22 | -82 | -16 |  |  |  |  |  |  |  |
| **AD < HC** |  |  |  |  |  |  |  |  |  |  |  |  |  |
| / |  |  |  |  |  |  | R PCUN | 217 | 7 | 4.38* | 6 | -54 | 60 |
|  |  |  |  |  |  |  | L/R CUN | 514 | 18 | 5.18* | 0 | -82 | 38 |
|  |  |  |  |  |  |  | R LING | 179 | 19 | 4.79* | 22 | -58 | -4 |
|  |  |  |  |  |  |  | R MTG | 210 | 37 | 3.95* | 54 | -64 | 10 |
|  |  |  |  |  |  |  | L SPL | 115 | 7 | 4.36 | -34 | -52 | 64 |
|  |  |  |  |  |  |  | L SMG | 40 | 40 | 4.51 | -48 | -32 | 26 |
|  |  |  |  |  |  |  | L MTG | 79 | 37 | 4.30 | -42 | -66 | 14 |
|  |  |  |  |  |  |  | R FFG | 48 | 37 | 4.25 | 46 | -52 | -24 |
|  |  |  |  |  |  |  | R PostCG | 158 | 3/7 | 3.96 | 26 | -38 | 58 |
|  |  |  |  |  |  |  | L PostCG | 41 | 43 | 3.74 | -56 | -14 | 16 |
|  |  |  |  |  |  |  | L LING | 65 | 18 | 3.93 | -12 | -70 | -8 |
|  |  |  |  |  |  |  | L LING | 41 |  | 4.22 | -18 | -60 | -8 |
|  |  |  |  |  |  |  | L CAL | 120 | 18 | 4.06 | -6 | -78 | 10 |

Abbreviations: ACC = Anterior cingulate cortex; AD = Alzheimer’s disease; aMCI = Amnestic mild cognitive impairment; AMYG = Amygdala; ANG = Angular gyrus; CUN = Cuneus; FFG = Fusiform gyrus; GSR = Global signal regression; HC = Healthy controls; HIP = Hippocampus; ICA = Independent component analysis; IFGoperc = Opercular part of the inferior frontal gyrus; INS = Insula; IOG = Inferior occipital gyrus; IPL = Inferior parietal lobule; ITG = Inferior temporal gyrus; L = Left; LING = Lingual gyrus; MCC = Mid-cingulate cortex; MOG = Middle occipital gyrus; MTG = Middle temporal gyrus; ORBmid = Middle frontal gyrus, orbital part; PAL = Pallidum; PARC = Paracentral lobule; PCC = Posterior cingulate cortex; PCUN = Precuneus; PHG = Parahippocampal gyrus; PostCG = Post central gyrus; PreCG = Precentral gyrus; PUT = Putamen; R = Right; ROL = Rolandic operculum; SMA = Supplementary motor area; SMG = Supramarginal gyrus; SOG = Superior occipital gyrus; SPL = Superior parietal lobule; STG = Superior temporal gyrus; VLPFC = Ventrolateral prefrontal cortex.

*Clusters surviving p < 0.05 family wise error (FWE) correction on the cluster level, with a previous height threshold of p < 0.001 (uncorrected).

# Supplementary Table 2 Slow4 BOLD variability comparisons between AD, aMCI, and HC with GSR or ICA-based denoising.

| **GSR** | | | | | | | **ICA** | | | | | | |
| --- | --- | --- | --- | --- | --- | --- | --- | --- | --- | --- | --- | --- | --- |
| **Regions** | **k** | **BA** | **Z** | **MNI coordinates** | | | **Regions** | **k** | **BA** | **Z** | **MNI coordinates** | | |
|  |  |  |  | x | y | z |  |  |  |  | x | y | z |
| **Main effect of group** |  |  |  |  |  |  |  |  |  |  |  |  |  |
| L/R PCUN/ ANG/ MOG/ CUN | 6533 | 7/19/  31/39/40 | 6.98* | 0 | -72 | 36 | ANG | 214 | 39/40 | 4.92* | -44 | -60 | 36 |
|  |  |  |  |  |  |  | L/R PCUN | 153 | 7 | 3.97 | 4 | -66 | 36 |
| R INS | 111 | 13 | 4.50* | 38 | -14 | -10 | R INS | 74 | 13 | 4.11 | 30 | 20 | 0 |
| L VMPFC | 60 | 10 | 4.61 | -8 | 58 | 22 |  |  |  |  |  |  |  |
| R VMPFC | 47 | 10 | 4.57 | 6 | 62 | 10 |  |  |  |  |  |  |  |
| ORBinf/INS | 51 | 47 | 4.16 | 26 | 14 | -22 |  |  |  |  |  |  |  |
| R PostCG | 40 |  | 5.22 | 58 | -8 | 26 |  |  |  |  |  |  |  |
| CAL | 144 | 17 | 4.35 | -10 | -88 | 0 |  |  |  |  |  |  |  |
| **aMCI > AD** |  |  |  |  |  |  |  |  |  |  |  |  |  |
| L/R PCUN/ MOG/ ANG/ CUN | 8576 | 7/19/31/39/40 | 7.06* | 0 | -72 | 36 | L ANG | 345 | 39/40 | 5.16* | -44 | -60 | 36 |
|  |  |  |  |  |  |  | L/R PCUN | 133 | 7 | 4.14 | -10 | -70 | 34 |
| L/R SFGmed | 293 | 10 | 4.36* | -4 | 54 | 8 |  |  |  |  |  |  |  |
| L CAL | 288 | 17 | 4.82* | -10 | -88 | 0 |  |  |  |  |  |  |  |
| R CAL | 79 | 17 | 4.18 | 14 | -88 | 4 |  |  |  |  |  |  |  |
| L SFG | 93 | 8 | 4.52 | -24 | 34 | 46 |  |  |  |  |  |  |  |
| L/R PARC | 126 | 6 | 3.64 | 10 | -22 | 74 |  |  |  |  |  |  |  |
| L DLPFC | 90 |  | 5.22 | -44 | 16 | 48 |  |  |  |  |  |  |  |
| R MCC | 63 | 23 | 4.34 | 6 | -32 | 34 |  |  |  |  |  |  |  |
| R PreCG | 70 | 6 | 4.55 | 40 | -18 | 66 |  |  |  |  |  |  |  |
| L LING | 49 | 18 | 4.33 | -22 | -78 | -14 |  |  |  |  |  |  |  |
| **aMCI < AD** |  |  |  |  |  |  |  |  |  |  |  |  |  |
| R PHG/HIP | 111 |  | 4.21* | 24 | -30 | -6 | / |  |  |  |  |  |  |
| L HIP/PUT | 65 |  | 4.01 | -34 | -16 | -4 |  |  |  |  |  |  |  |
| R OLF/ORBinf | 62 | 47 | 4.23 | 26 | 14 | -22 |  |  |  |  |  |  |  |
| L ORBinf/INS | 60 |  | 4.18 | -28 | 24 | -12 |  |  |  |  |  |  |  |
| R VLPFC | 47 |  | 4.76 | 46 | 32 | 10 |  |  |  |  |  |  |  |
| R PAL | 44 |  | 4.48 | 22 | -4 | -6 |  |  |  |  |  |  |  |
| **aMCI > HC** |  |  |  |  |  |  |  |  |  |  |  |  |  |
| L PCUN/CUN/MOG/ ANG | 4218 | 7/19/31/39/40 | 5.80* | -36 | -56 | 52 |  |  |  |  |  |  |  |
| L IPL/ANG | 478 | 40 | 5.22 | -46 | -56 | 32 |  |  |  |  |  |  |  |
|  |  |  |  |  |  |  | R INS | 200 | 13 | 4.37* | 30 | 20 | 0 |
| R PostCG | 71 |  | 5.69 | 58 | -8 | 26 |  |  |  |  |  |  |  |
| L PCUN | 48 |  | 4.01 | -14 | -48 | 64 |  |  |  |  |  |  |  |
| R MOG | 80 | 19 | 5.26 | 30 | -96 | 14 |  |  |  |  |  |  |  |
| L/R VMPFC | 125 | 10 | 4.84 | -8 | 58 | 22 |  |  |  |  |  |  |  |
| L VLPFC/DLPFC | 75 |  | 4.05 | -48 | 16 | 34 |  |  |  |  |  |  |  |
| L DLPFC | 49 |  | 3.88 | -40 | 34 | 32 |  |  |  |  |  |  |  |
| L MTG | 44 | 21 | 4.47 | -66 | -22 | -6 |  |  |  |  |  |  |  |
| R IPL/ANG | 67 |  | 4.46 | 54 | -52 | 38 |  |  |  |  |  |  |  |
| R IPL | 68 | 40 | 4.03 | 50 | -48 | 50 |  |  |  |  |  |  |  |
| **aMCI < HC** |  |  |  |  |  |  |  |  |  |  |  |  |  |
| R INS | 288 | 13 | 4.72* | 42 | -8 | 6 | / |  |  |  |  |  |  |
| R INS/ORBinf | 160 | 13/47 | 4.40* | 38 | 10 | -2 |  |  |  |  |  |  |  |
| L INS/TmP | 79 | 38 | 3.66 | -38 | 6 | -20 |  |  |  |  |  |  |  |
| **AD < HC** |  |  |  |  |  |  |  |  |  |  |  |  |  |
| R Cerebellum/FFG | 55 |  | 5.29 | 40 | -56 | -24 |  |  |  |  |  |  |  |
|  |  |  |  |  |  |  | L ANG | 275 | 39 | 4.60* | -42 | -60 | 38 |
|  |  |  |  |  |  |  | L/R PCUN | 562 | 7 | 4.33* | 4 | -66 | 36 |
|  |  |  |  |  |  |  | R PCL/MCC | 163 |  | 4.50 | 0 | -28 | 52 |
|  |  |  |  |  |  |  | L SPL/IPL | 70 |  | 4.11 | -36 | -48 | 60 |
|  |  |  |  |  |  |  | L SPL | 121 | 7 | 4.08 | -20 | -64 | 56 |
| **AD > HC** |  |  |  |  |  |  |  |  |  |  |  |  |  |
| / |  |  |  |  |  |  | R ORBinf | 107 | 47 | 3.90 | 46 | 40 | -16 |
|  |  |  |  |  |  |  | R INS | 79 |  | 4.14 | 30 | 20 | 0 |

Abbreviations: AD = Alzheimer’s disease; aMCI = Amnestic mild cognitive impairment; ANG = Angular gyrus; CAL = Calcarine sulcus; CUN = Cuneus; DLPFC = Dorsolateral prefrontal cortex; FFG = Fusiform gyrus; GSR = Global signal regression; HC = Healthy controls; HIP = Hippocampus; ICA = Independent component analysis; INS = Insula; IPL = Inferior parietal lobule; L = Left; LING = Lingual gyrus; MCC = Mid-cingulate cortex; MOG = Middle occipital gyrus; MTG = Middle temporal gyrus; OLF = Olfactory cortex; ORBinf = Inferior frontal gyrus, orbital part; PAL = Pallidum; PCL = paracentral lobule; PCUN = Precuneus; PHG = Parahippocampal gyrus; PostCG = Postcentral gyrus; PreCG = Precentral gyrus; PUT = Putamen; R = Right; SFGmed = Medial part of the superior frontal gyrus; TmP = Temporal pole; VLPFC = Ventrolateral prefrontal cortex; VMPFC = Ventromedial prefrontal cortex.

*Clusters surviving p < 0.05 family wise error (FWE) correction on the cluster level, with a previous height threshold of p < 0.001 (uncorrected).

**Supplementary Table 3** Whole band BOLD SD (z-score) comparisons between AD, aMCI, and HC with GSR or ICA-based denoising.

| **GSR** | | | | | | | **ICA** | | | | | | |
| --- | --- | --- | --- | --- | --- | --- | --- | --- | --- | --- | --- | --- | --- |
| **Regions** | **k** | **BA** | **Z** | **MNI coordinates** | | | **Regions** | **k** | **BA** | **Z** | **MNI coordinates** | | |
|  |  |  |  | x | y | z |  |  |  |  | x | y | z |
| **Main effect of group** |  |  |  |  |  |  |  |  |  |  |  |  |  |
| L LING/CAL | 119 | 18 | 5.23* | -22 | -62 | 0 | L LING/CAL | 233 | 18 | 5.72* | -22 | -58 | 0 |
|  |  |  |  |  |  |  | R LING/CAL | 217 | 18/30 | 5.28* | 12 | -48 | 4 |
|  |  |  |  |  |  |  | L SOG/CUN | 247 | 19 | 5.02* | -14 | -86 | 44 |
|  |  |  |  |  |  |  | R DLPFC/SFG | 720 | 8 | 4.85* | 26 | 26 | 40 |
|  |  |  |  |  |  |  | L CAL/LING | 137 | 17/18 | 4.12 | -6 | -104 | -8 |
|  |  |  |  |  |  |  | L MOG | 103 | 19 | 4.54 | -50 | -82 | 2 |
| L THA | 225 |  | 4.50* | -8 | -16 | 2 |  |  |  |  |  |  |  |
| L PreCG | 46 | 6/8 | 4.51 | -50 | 8 | 46 |  |  |  |  |  |  |  |
| L PostCG | 68 | 3 | 4.49 | -34 | -26 | 44 |  |  |  |  |  |  |  |
| L/R SMA | 62 |  | 4.04 | 4 | 4 | 46 |  |  |  |  |  |  |  |
| R Cerebellum | 96 |  | 3.96 | 28 | -66 | -56 |  |  |  |  |  |  |  |
| R ACC/MPFC | 75 | 32 | 3.94 | 6 | 50 | 12 |  |  |  |  |  |  |  |
| L MTG | 61 | 21 | 3.90 | -70 | -28 | -6 |  |  |  |  |  |  |  |
|  |  |  |  |  |  |  | R MTG | 48 | 19/39 | 4.01 | 58 | -66 | 10 |
|  |  |  |  |  |  |  | L/R PCUN | 68 |  | 3.96 | 0 | -60 | 66 |
| **aMCI > AD** |  |  |  |  |  |  |  |  |  |  |  |  |  |
| L PostCG | 160 | 3/4 | 5.00* | -34 | -26 | 44 | / |  |  |  |  |  |  |
| L MTG | 369 | 21 | 4.34* | -46 | -32 | -8 |  |  |  |  |  |  |  |
| L THA | 152 |  | 4.02* | -12 | -22 | 6 |  |  |  |  |  |  |  |
| L PreCG | 93 | 6/8 | 4.47 | -50 | 8 | 46 |  |  |  |  |  |  |  |
| L PCUN/PCC | 130 | 31 | 4.24 | -8 | -54 | 28 |  |  |  |  |  |  |  |
| L MTG | 58 | 21 | 3.93 | -58 | -54 | 2 |  |  |  |  |  |  |  |
| L ROL | 45 | 6 | 3.80 | -60 | 8 | 0 |  |  |  |  |  |  |  |
| **aMCI < AD** |  |  |  |  |  |  |  |  |  |  |  |  |  |
| R/L Cerebellum | 225 |  | 4.50* | 8 | -78 | -38 |  |  |  |  |  |  |  |
| R Cerebellum | 224 |  | 4.34* | 28 | -66 | -56 |  |  |  |  |  |  |  |
| R/L Cerebellum | 181 |  | 4.18 | 10 | -82 | -24 |  |  |  |  |  |  |  |
| L Cerebellum | 99 |  | 3.88 | -26 | -60 | -58 |  |  |  |  |  |  |  |
| R PostCG | 74 | 3 | 4.25 | 36 | -36 | 58 |  |  |  |  |  |  |  |
| R ORBsup/SFG | 96 | 10 | 4.11 | 24 | 52 | 0 | R DLPFC/SFG | 295 | 8 | 3.88 | 22 | 20 | 46 |
| L/R SMA | 71 |  | 4.00 | 0 | 4 | 62 |  |  |  |  |  |  |  |
|  |  |  |  |  |  |  | L SPL/SOG | 123 | 7/19 | 3.77 | -24 | -76 | 42 |
| **aMCI > HC** |  |  |  |  |  |  |  |  |  |  |  |  |  |
| / |  |  |  |  |  |  | / |  |  |  |  |  |  |
| **aMCI < HC** |  |  |  |  |  |  |  |  |  |  |  |  |  |
| L LING/CAL | 237 | 18/30 | 5.49* | -22 | -62 | 0 | L CAL/LING | 251 | 18/30 | 5.42* | -22 | -58 | 0 |
|  |  |  |  |  |  |  | R CAL/LING | 355 | 18/30 | 5.37* | 12 | -48 | 4 |
|  |  |  |  |  |  |  | L CAL | 118 | 17/18 | 4.78 | -6 | -104 | -8 |
|  |  |  |  |  |  |  | L CAL/CUN | 180 | 18/23/31 | 3.97 | -4 | -60 | 20 |
| L MOG/SOG | 86 | 19 | 4.33 | -22 | -84 | 20 |  |  |  |  |  |  |  |
| R MOG | 91 | 19 | 4.27 | 44 | -86 | 4 | R MOG | 198 | 19 | 4.44 | 34 | -94 | 12 |
| L SOG/CUN | 87 | 19 | 4.09 | -10 | -86 | 44 | L SOG/CUN | 401 | 19 | 5.08* | -14 | -86 | 44 |
|  |  |  |  |  |  |  | R CUN | 139 | 19 | 3.99 | 8 | -82 | 40 |
| R LING/Cerebellum | 64 | 18/19 | 4.05 | 40 | -84 | -18 |  |  |  |  |  |  |  |
|  |  |  |  |  |  |  | R PCUN | 69 | 7 | 4.25 | 2 | -60 | 66 |
| **AD < HC** |  |  |  |  |  |  |  |  |  |  |  |  |  |
| L LING/CAL | 208 | 18/19/30 | 4.87* | -22 | -62 | 0 | L LING/CAL | 730 | 18/23/30 | 5.85* | -22 | -58 | 0 |
|  |  |  |  |  |  |  | L CAL/LING  /MOG | 407 | 17/18 | 5.38* | -6 | -104 | -8 |
|  |  |  |  |  |  |  | R LING/CAL | 332 | 18/19/30 | 5.18* | 12 | -50 | 4 |
|  |  |  |  |  |  |  | L SOG/PCUN  /CUN | 423 | 7/19 | 5.15* | -12 | -86 | 44 |
|  |  |  |  |  |  |  | R MOG/MTG | 376 | 19 | 4.46* | 58 | -66 | 10 |
| L THA | 301 |  | 4.85* | -8 | -18 | 8 | L THA | 87 |  | 4.15 | -8 | -18 | 8 |
| L/R PCUN | 42 |  | 4.91 | 0 | -74 | 58 |  |  |  |  |  |  |  |
| L PCUN/CAL | 75 | 23 | 4.05 | -2 | -64 | 16 |  |  |  |  |  |  |  |
| L PreCG | 52 | 6/8 | 4.59 | -50 | 8 | 48 |  |  |  |  |  |  |  |
| R THA | 59 |  | 4.13 | 10 | -14 | 0 |  |  |  |  |  |  |  |
| L MTG | 50 | 21 | 3.99 | -70 | -32 | -8 | L MOG/MTG | 254 | 19/37/39 | 5.04* | -50 | -82 | 2 |
| L MTG | 53 | 21 | 3.89 | -60 | -58 | 2 |  |  |  |  |  |  |  |
|  |  |  |  |  |  |  | R CUN/SOG | 107 | 19 | 4.00 | 8 | -90 | 24 |
|  |  |  |  |  |  |  | L ROL | 60 | 22 | 4.59 | -66 | -6 | 10 |
|  |  |  |  |  |  |  | R ITG | 90 | 20/37 | 4.55 | 56 | -56 | -18 |
| **AD > HC** |  |  |  |  |  |  |  |  |  |  |  |  |  |
| L/R ACC/MPFC | 236 | 10/32 | 4.48* | 6 | 50 | 12 |  |  |  |  |  |  |  |
|  |  |  |  |  |  |  | R DLPFC/SFG/ MPFC | 2058 | 8/9/32 | 5.32* | 26 | 26 | 40 |
| L/R SMA/MCC | 168 | 24/32 | 4.18* | -4 | 12 | 46 |  |  |  |  |  |  |  |
|  |  |  |  |  |  |  | L IPL | 122 | 40 | 4.31 | -42 | -52 | 38 |

Abbreviations: AD = Alzheimer’s disease; aMCI = Amnestic mild cognitive impairment; ANG = Angular gyrus; CAL = Calcarine sulcus; CUN = Cuneus; DLPFC = Dorsolateral prefrontal cortex; FFG = Fusiform gyrus; GSR = Global signal regression; HC = Healthy controls; HIP = Hippocampus; ICA = Independent component analysis; IPL = Inferior parietal lobule; ITG = Inferior temporal gyrus; L = Left; LING = Lingual gyrus; MCC = Mid-cingulate cortex; MOG = Middle occipital gyrus; MPFC = Medial prefrontal cortex; MTG = Middle temporal gyrus; ORBsup = Superior frontal gyrus, orbital part; PCC = Posterior cingulate cortex; PCUN = Precuneus; PostCG = Postcentral gyrus; PreCG = Precentral gyrus; PUT = Putamen; R = Right; ROL = Rolandic operculum; SFG = Superior frontal gyrus; SMA = Supplementary motor area; SOG = Superior occipital gyrus; THA = Thalamus.

*Clusters surviving p < 0.05 family wise error (FWE) correction on the cluster level, with a previous height threshold of p < 0.001 (uncorrected).

# Supplementary Table 4 Correlations between BOLD variability and baseline global cognition, hippocampal volume and global cognitive decline over a 2-year follow-up^a^ in all patients using both GSR or ICA-based denoising.

| Region |  | GSR | | |  | ICA | | |
| --- | --- | --- | --- | --- | --- | --- | --- | --- |
|  |  | Global cognition^b^ | HIP | Global cognitive decline |  | Global cognition^b^ | HIP | Global cognitive decline |
| **Slow4** |  |  |  |  |  |  |  |  |
| **aMCI > HC** | L PCUN/CUN/  MOG/ANG | 0.35*****^+^** | 0.26*****^+^** | 0.21* |  | / | / | / |
|  | R INS | / | / | / |  | 0.006 | 0.014 | -0.01 |
| **HC > aMCI** | R INS | -0.12 | -0.02 | 0.03 |  | / | / | / |
|  | R INS/ORBinf | -0.23****^+^** | -0.14 | 0.09 |  | / | / | / |
| **HC > AD** | L ANG | / | / | / |  | 0.22****^+^** | 0.13 | 0.12 |
|  | L/R PCUN | / | / | / |  | 0.19****^+^** | 0.21****^+^** | 0.21* |
| **Slow5** |  |  |  |  |  |  |  |  |
| **HC > aMCI** | L INS/ROL | -0.08 | -0.09 | -0.02 |  | -0.10 | -0.07 | 0.01 |
|  | R INS/IFGoperc | -0.12 | -0.11 | -0.11 |  | / | / | / |
|  | R PUT | -0.25*****^+^** | -0.11 | -0.14 |  | / | / | / |
|  | L PHG | -0.21****^+^** | -0.11 | 0.08 |  | / | / | / |
|  | L Cerebellum | -0.15* | -0.09 | -0.08 |  | / | / | / |
| **aMCI > HC** | L/R PCUN/ANG/  CUN/MOG/  SOG (GSR) or  L ANG (ICA) | 0.32*****^+^** | 0.13 | 0.03 |  | -0.001 | -0.06 | -0.02 |
| **HC > AD** | R PCUN | / | / | / |  | 0.10 | -0.04 | 0.10 |
|  | L/R CUN | / | / | / |  | 0.17* | 0.06 | 0.11 |
|  | R LING | / | / | / |  | 0.26*****^+^** | 0.15* | 0.13 |
|  | R MTG | / | / | / |  | 0.24*****^+^** | 0.11 | 0.18 |

Values represent correlation coefficient (r). ***p ≤ 0.001, **p < 0.01, *p < 0.05. **^+^**Correlations surviving multiple comparisons correction (p < 0.05 corrected).

^a^Global cognitive decline is defined as the difference in global cognition between the end of year 2 and baseline (year 2 minus baseline). ^b^One patient with Alzheimer’s disease does not have Global cognition data. Abbreviations: ANG = Angular gyrus; CAL = Calcarine sulcus; CUN = Cuneus; GSR = Global signal regression; HIP = Hippocampal volume; ICA = Independent component analysis; IFGoperc = Opercular part of the inferior frontal gyrus; INS = Insula; L = Left; LING = Lingual gyrus; MOG = Middle occipital gyrus; MTG = Middle temporal gyrus; PCUN = Precuneus; PHG = Parahippocampal gyrus; PUT =Putamen; R = Right.

**Supplementary Table 5** Group comparisons of demographic and neuropsychological features between included and excluded AD.

|  | Included AD  (n = 96) | Excluded AD  (n = 28) | t/ χ^2^ | *p* |
| --- | --- | --- | --- | --- |
| Age, yrs | 74.43 (7.13) | 77.36 (5.88) | 1.98 | 0.05 |
| Male/Female | 37/59 | 7/21 | 1.74 | 0.188 |
| Handedness, R/L | 94/2 | 27/1 | 0.20 | 0.652 |
| Ethnicity, C/non-C | 74/22 | 21/7 | 0.05 | 0.819 |
| Education, yrs | 4.92 (4.93) | 4.11 (4.32) | -0.79 | 0.434 |
| CDR-SOB | 6.70 (2.73) | 8.63 (3.96) | 2.95 | 0.004* |
| MMSE | 16.10 (4.40) | 14.25 (5.83) | -1.81 | 0.072 |
| MoCA | 11.13 (4.67) | 8.0 (4.51) | -3.14 | 0.002* |

Values represent mean (s.d). Abbreviations: AD = Alzheimer’s disease; C/non-C = Chinese/non-Chinese; CDR-SOB = Clinical Dementia Rating Scale Sum of Boxes; MMSE = Mini-Mental State Examination; MoCA = Montreal Cognitive Assessment; R/L = Right/left. *p < 0.05.


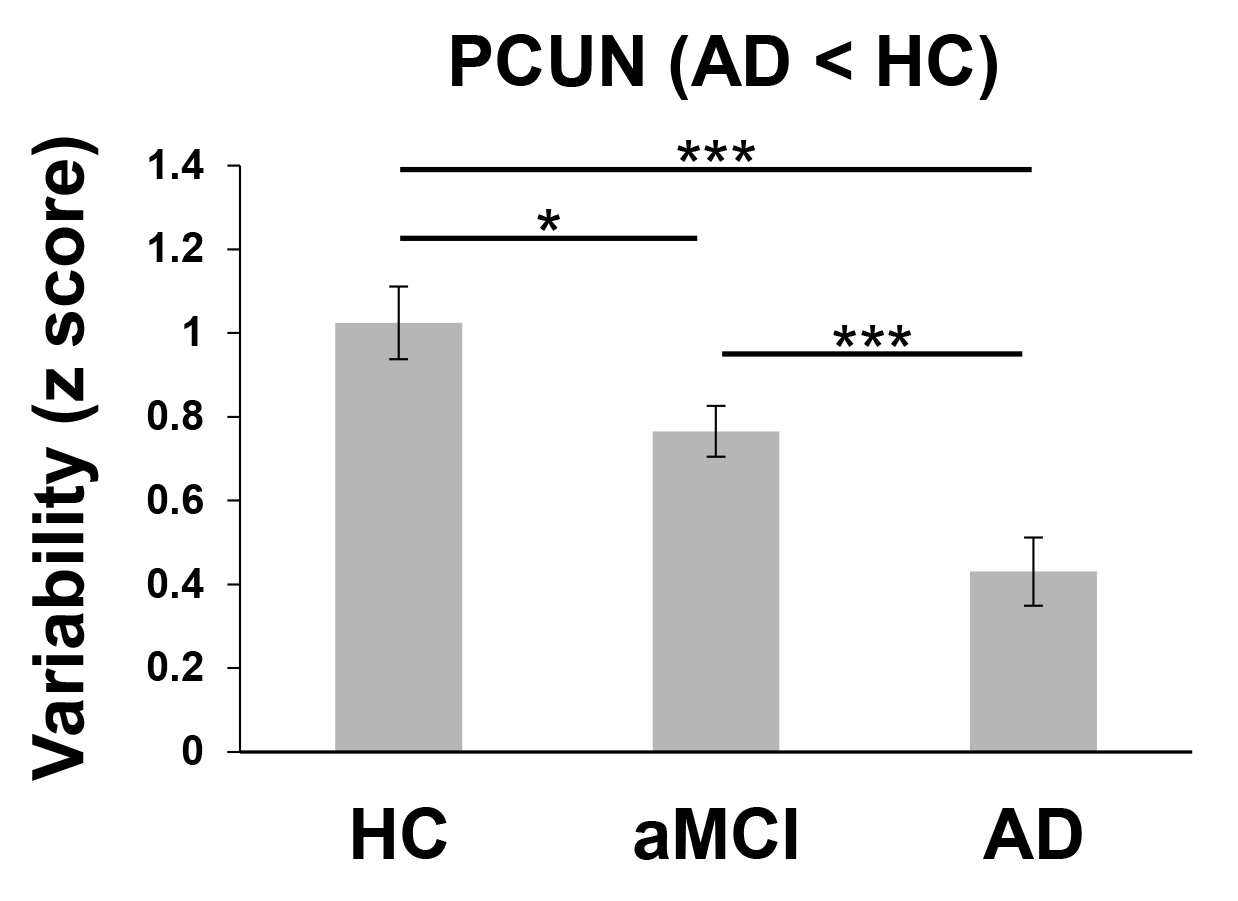


# Supplementary Figure S1. Lower DMN variability in AD and aMCI compared with HC at slow4 with ICA-based denoising. Bar charts represented mean variability in the PCUN based on the comparison between AD and HC (error bar = one standard error). HC showed the highest variability and AD the lowest, with aMCI displaying an intermediate level of variability. Results were obtained at p < 0.05 family wise error (FWE) correction on the cluster level, with a previous height threshold of p < 0.001 (uncorrected). Abbreviations: AD = Alzheimer’s disease; aMCI = Amnestic mild cognitive impairment; CUN = Cuneus; HC = Healthy controls; ICA = Independent component analysis; PCUN = Precuneus. ***p ≤ 0.001, **p ≤ 0.01, *p < 0.05.

#
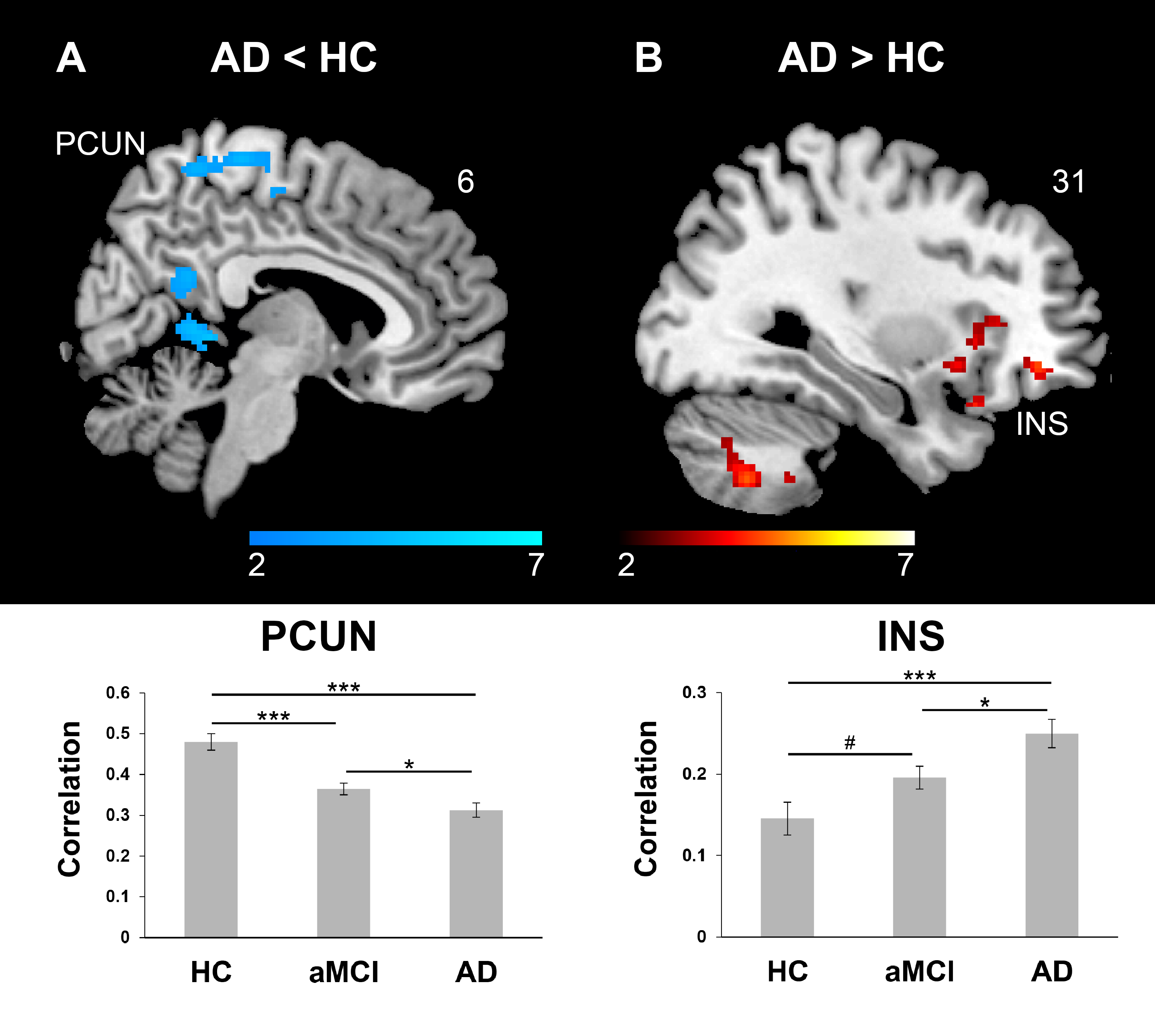


**Supplementary Figure S2.** **Divergent correlations of global signal with slow4 time series in the default mode network and salience network in AD compared with HC.** Global signal showed weaker correlation with the time course in the default mode network (PCUN) (A), and stronger correlation with the time course in the salience network (INS) (B) in AD compared with HC, with aMCI showing an intermediate level of correlation. Results were obtained at p < 0.05 family wise error (FWE) correction on the cluster level, with a previous height threshold of p < 0.001 (uncorrected), superimposing on the MNI brain template. The cluster of the insula (B) was observed only with a cluster-level threshold of p < 0.05 (uncorrected), with a previous voxel-defining threshold of p < 0.001 (uncorrected) and a cluster extent threshold of k > 40. Colour bar represents T value. Moreover, plotting of correlation between global signal and time series in the clusters from group comparisons is displayed in the corresponding bottom panels. Error bar represents one standard error. Abbreviations: AD = Alzheimer’s disease; aMCI = Amnestic mild cognitive impairment; HC = Healthy controls; INS = Insula; PCUN = Precuneus.

***p < 0.001, *p < 0.05, ^#^p = 0.055.

#
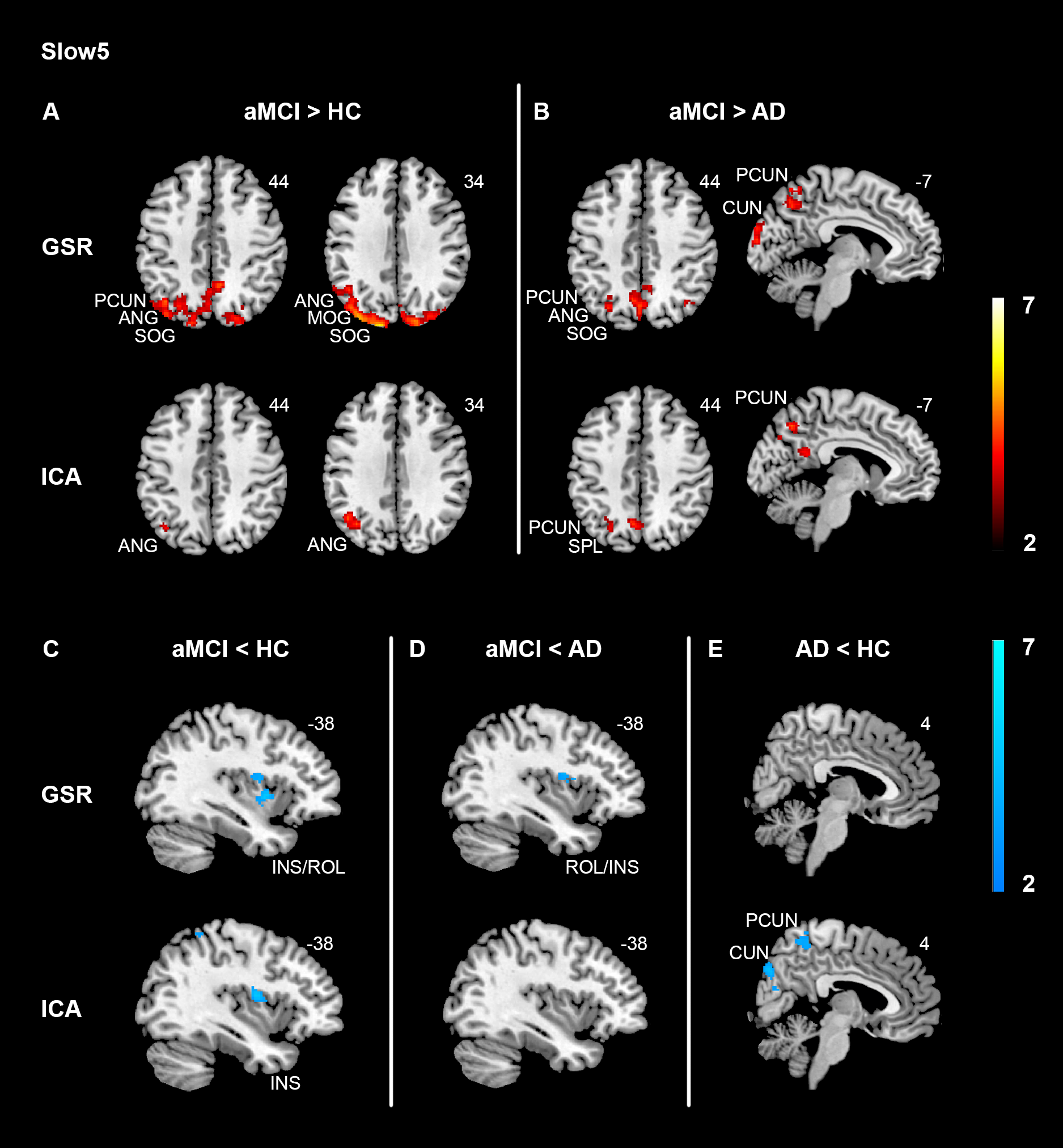


# Supplementary Figure S3. Divergent slow5 hemodynamic variability changes in the default mode and the salience networks in amnestic MCI and AD controlling for motion. After further controlling for motion, comparable results were obtained. Specifically, using both GSR and ICA-based denoising methods, aMCI showed higher variability in the default mode network compared with HC (A) and AD (B), and lower variability in the salience network (INS) compared with HC (C). Specific to data denoising approaches, ICA-based denoising revealed lower variability in the default mode network (PCUN) in AD compared with HC (E, bottom panel), while GSR method showed lower variability in the salience network (INS) in aMCI compared with AD (D, top panel). Results were obtained at p < 0.05 family wise error (FWE) correction on the cluster level, with a previous height threshold of p < 0.001, superimposing on the MNI brain template. We also reported results with a less stringent cluster-level threshold of p < 0.05 (uncorrected, k > 40), with a previous height threshold of p < 0.001 (for the angular gyrus at aMCI > HC [A, bottom panel] and the insula at aMCI < HC [C, bottom panel] with ICA-based denoising). Colour bar represents T value. Abbreviations: AD = Alzheimer’s disease; aMCI = Amnestic mild cognitive impairment; ANG = Angular gyrus; CUN = Cuneus; GSR = Global signal regression; HC = Healthy controls; ICA = Independent component analysis; INS = Insula; PCUN = Precuneus; ROL = Rolandic operculum; SOG = Superior occipital gyrus. (Color should be used for this figure in print)

#
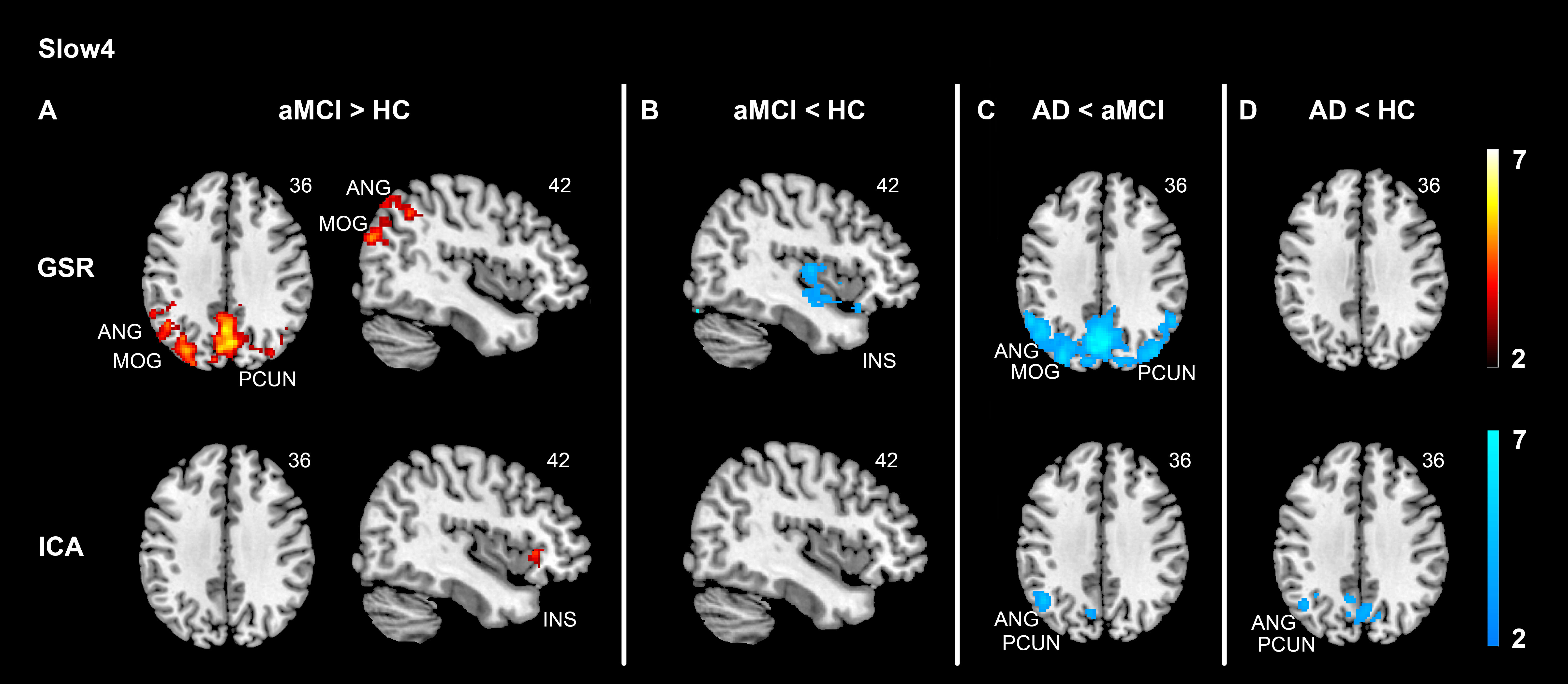


# Supplementary Figure S4. Divergent slow4 hemodynamic variability changes in the default mode and the salience networks in amnestic MCI and AD controlling for motion. Comparable results were obtained after further controlling for motion. Specifically, compared with HC, GSR approach revealed higher variability in the default mode network (A, top panel) while lower variability in the salience network (B, top panel) in aMCI. However, after ICA-based denoising, there was higher variability in the salience network in aMCI than in HC (A, bottom panel). Moreover, AD showed lower variability in the default mode network (PCUN, ANG) compared with HC after ICA-based denoising (D, bottom panel), which was absent for the GSR approach (D, top panel). Across both data denoising methods, AD showed lower variability in the default mode network compared with aMCI (C). Results were obtained at p < 0.05 family wise error (FWE) correction on the cluster level, with a previous height threshold of *p* < 0.001, superimposing on the MNI brain template. Colour bar represents T value. Abbreviations: AD = Alzheimer’s disease; aMCI = Amnestic mild cognitive impairment; ANG = Angular gyrus; GSR = Global signal regression; HC = Healthy controls; ICA = Independent component analysis; INS = Insula; MOG = Middle occipital gyrus; PCUN = Precuneus. (Color should be used for this figure in print)


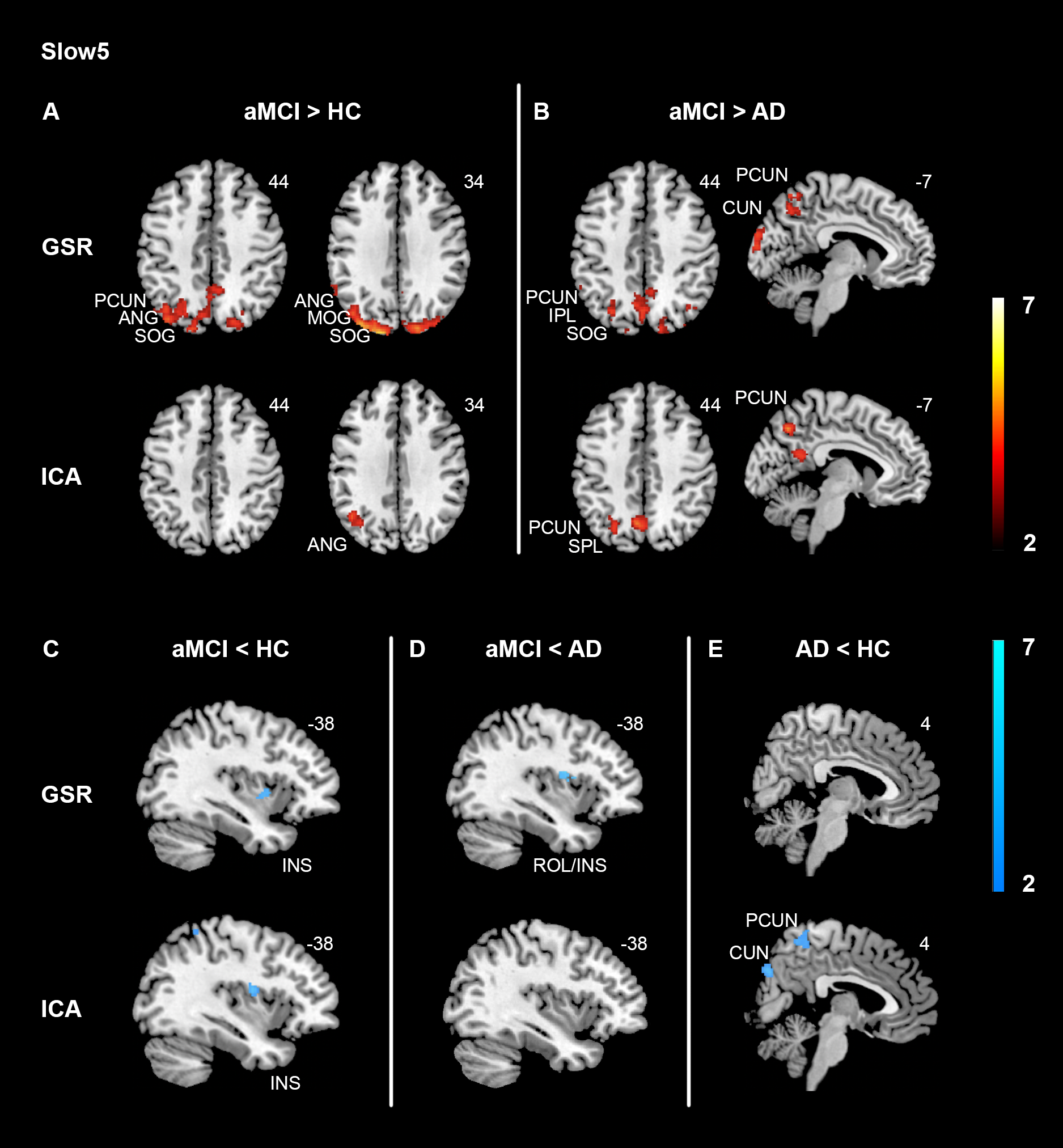


# Supplementary Figure S5. Divergent slow5 hemodynamic variability changes in the default mode and the salience networks in amnestic MCI and AD controlling for cerebrovascular disease. After further controlling for presence of significant CeVD, comparable results were obtained. Specifically, using both GSR and ICA-based denoising methods, aMCI showed higher variability in the default mode network compared with HC (A) and AD (B), and lower variability in the salience network (INS) compared with HC (C). Specific to data denoising approaches, ICA-based denoising revealed lower variability in the default mode network (PCUN) in AD compared with HC (E, bottom panel), while GSR method showed lower variability in the salience network (INS) in aMCI compared with AD (D, top panel). Results were obtained at p < 0.05 family wise error (FWE) correction on the cluster level, with a previous height threshold of p < 0.001, superimposing on the MNI brain template. We also reported results with a less stringent cluster-level threshold of p < 0.05 (uncorrected, k > 40), with a previous height threshold of p < 0.001 (for the INS at aMCI < HC [C] for both GSR and ICA-based denoising]). Colour bar represents T value. Abbreviations: AD = Alzheimer’s disease; aMCI = Amnestic mild cognitive impairment; ANG = Angular gyrus; CeVD = Cerebrovascular disease; CUN = Cuneus; GSR = Global signal regression; HC = Healthy controls; ICA = Independent component analysis; INS = Insula; IPL = Inferior parietal lobule; PCUN = Precuneus; ROL = Rolandic operculum; SOG = Superior occipital gyrus. (Color should be used for this figure in print)


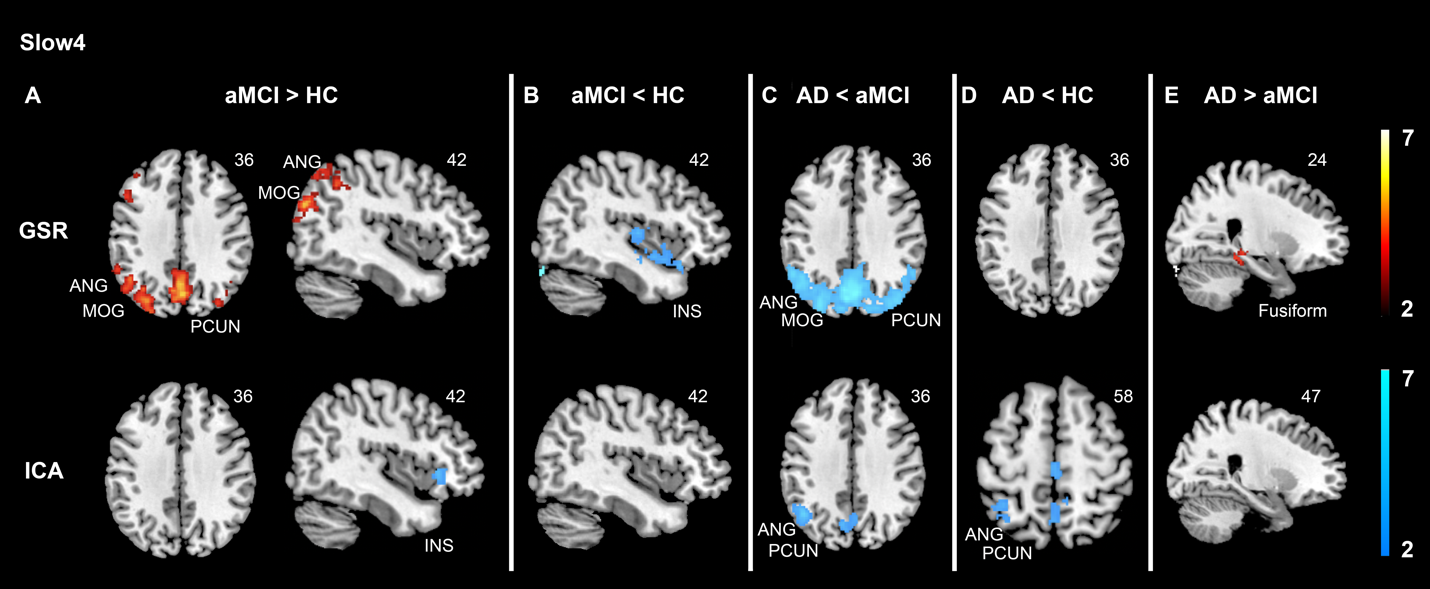


# Supplementary Figure S6. Divergent slow4 hemodynamic variability changes in the default mode and the salience networks in amnestic MCI and AD controlling for cerebrovascular disease. Comparable results were obtained after further controlling for presence of significant CeVD. Specifically, compared with HC, GSR approach revealed higher variability in the default mode network (A, top panel) while lower variability in the salience network (B, top panel) in aMCI. However, after ICA-based denoising, there was higher variability in the salience network in aMCI than in HC (A, bottom panel). Moreover, AD showed lower variability in the default mode network (PCUN, ANG) compared with HC after ICA-based denoising (D, bottom panel), which was absent for the GSR approach (D, top panel). In addition, AD had higher variability in the fusiform gyrus compared with aMCI with GSR approach (E, top panel), which was absent for the ICA-based denoising (E, bottom panel). Across both data denoising methods, AD showed lower variability in the default mode network compared with aMCI (C). Results were obtained at p < 0.05 family wise error (FWE) correction on the cluster level, with a previous height threshold of *p* < 0.001, superimposing on the MNI brain template. Colour bar represents T value. Abbreviations: AD = Alzheimer’s disease; aMCI = Amnestic mild cognitive impairment; ANG = Angular gyrus; GSR = Global signal regression; HC = Healthy controls; ICA = Independent component analysis; INS = Insula; MOG = Middle occipital gyrus; PCUN = Precuneus. (Color should be used for this figure in print)

**REFERENCES**

1. Chong, J. S. X. *et al.* Influence of cerebrovascular disease on brain networks in prodromal and clinical Alzheimer’s disease. *Brain* **140**, 3012–3022 (2017).

2. van de Pol, L. A. *et al.* Hippocampal atrophy in Alzheimer disease: age matters. *Neurology* **66**, 236–8 (2006).

3. Jack, C. R., Jr. *et al.* Medial temporal atrophy on MRI in normal aging and very mild Alzheimer’s disease. *Neurology* **49**, 786–94 (1997).

4. Rajapakse, J. C., Giedd, J. N. & Rapoport, J. L. Statistical approach to segmentation of single-channel cerebral MR images. *IEEE Trans Med Imaging* **16**, 176–86 (1997).

5. Tzourio-Mazoyer, N. *et al.* Automated anatomical labeling of activations in SPM using a macroscopic anatomical parcellation of the MNI MRI single-subject brain. *Neuroimage* **15**, 273–89 (2002).
